# Ablation of BAF170 in Developing and Postnatal Dentate Gyrus Affects Neural Stem Cell Proliferation, Differentiation, and Learning

**DOI:** 10.1007/s12035-016-9948-5

**Published:** 2016-07-08

**Authors:** Tran Tuoc, Ekrem Dere, Konstantin Radyushkin, Linh Pham, Huong Nguyen, Anton B. Tonchev, Guoqiang Sun, Anja Ronnenberg, Yanhong Shi, Jochen F. Staiger, Hannelore Ehrenreich, Anastassia Stoykova

**Affiliations:** 1 Institute of Neuroanatomy, University Medical Center, Georg-August University Göttingen, Göttingen, Germany; 2 Max-Planck-Institute for Biophysical Chemistry, Göttingen, Germany; 3 DFG Center for Nanoscale Microscopy & Molecular Physiology of the Brain (CNMPB), Göttingen, Germany; 4 Clinical Neuroscience, Max Planck Institute of Experimental Medicine, Göttingen, Germany; 5 Department of Anatomy, Histology and Embryology, Medical University of Varna, Varna, Bulgaria; 6 Division of Stem Cell Biology Research, Department of Developmental and Stem Cell Biology, Cancer Center, Beckman Research Institute of City of Hope, 1500 E. Duarte Road, Duarte, CA 91010 USA

**Keywords:** SWI/SNF complex, BAF170, Hippocampus, Adult neuronal stem cells neurogenesis, Astrogenesis, Learning and memory

## Abstract

The BAF chromatin remodeling complex plays an essential role in brain development. However its function in postnatal neurogenesis in hippocampus is still unknown. Here, we show that in postnatal dentate gyrus (DG), the BAF170 subunit of the complex is expressed in radial glial-like (RGL) progenitors and in cell types involved in subsequent steps of adult neurogenesis including mature astrocytes. Conditional deletion of BAF170 during cortical late neurogenesis as well as during adult brain neurogenesis depletes the pool of RGL cells in DG, and promotes terminal astrocyte differentiation. These derangements are accompanied by distinct behavioral deficits, as reflected by an impaired accuracy of place responding in the Morris water maze test, during both hidden platform as well as reversal learning. Inducible deletion of BAF170 in DG during adult brain neurogenesis resulted in mild spatial learning deficits, having a more pronounced effect on spatial learning during the reversal test. These findings demonstrate involvement of BAF170-dependent chromatin remodeling in hippocampal neurogenesis and cognition and suggest a specific role of adult neurogenesis in DG in adaptive behavior.

**Electronic supplementary material:**

The online version of this article (doi:10.1007/s12035-016-9948-5) contains supplementary material, which is available to authorized users.

## Introduction

In the mammalian central nervous system (CNS), new neurons are generated throughout life in the subgranular zone (SGZ) of the DG in the hippocampus (Hi) where newly generated neurons play an essential role in certain types of learning and memory formation [1–7]. Generally, adult neurogenesis recapitulates main stages of neurogenesis in the developing brain: (1) proliferation of neural stem/progenitor cells (NSCs); (2) neuronal fate determination; (3) maturation and migration of neurons, and (4) functional integration of new neurons into existing neuronal circuits [1–5]. Recent evidence indicates that adult neurogenesis is regulated by transcription factors (TFs), hormones, neurotransmitters, composition of the cell niches as well as through exercise [1–5, 8]. Furthermore, epigenetic mechanisms were shown to exert relatively long-lasting biological effects on neurogenesis throughout life [8, 9]. Epigenetic control influences the accessibility of TFs to their consensus regulatory elements in downstream target genes by two main mechanisms: covalent histone modifications and non-covalent, energy-dependent chromatin modifications involving ATP-dependent chromatin remodeling complexes, such as SWI/SNF (SWItch/Sucrose NonFermentable) [8–10]. Vertebrate mSWI/SNF complexes contain two interchangeable core ATPase subunits (Brg1 or Brm), in combination with different BAF (Brg1/Brm-associated factor) subunits [10, 11]. Results from in vitro experiments have shown that the progressive transition from embryonic stem cells (ESCs) to neural progenitors, and later, toward post-mitotic neurons, is accompanied by subunit exchange within the BAF complexes [12, 13]. Interestingly, this process is accompanied by exchange of one BAF155 subunit in the ESC-specific BAF complex (esBAF) for BAF170 in the neural progenitor-specific chromatin-remodeling complex (npBAF) [12, 14], suggesting a possible important role of BAF170 in neurogenesis.

Results from our previous in vivo analyses of cortex-specific conditional BAF170 knockout (*BAF170cKO*) and overexpression *(BAF170cOE)* mice revealed that acting in a Brm-based BAF complex, BAF170 mediates chromatin control over the mode of cortical neurogenesis from the radial glial progenitors (RGPs), exerting a temporal repression of indirect neurogenesis from generated intermediate (IP) progenitors [15, 16]. Here, we present evidence that acting in another, Brg1-based BAF complex, BAF170 exerts a control of neurogenesis in the hippocampal niche for adult brain neurogenesis. Particularly, we found that postnatal conditional knockout of BAF170 expression causes a depletion of the pool of radial glia-like (RGL) cells and neuronal progenitors in SGZ of the DG as a consequence of premature generation of astrocytes. Furthermore, the impairment of the hippocampal postnatal neurogenesis in *BAF170cKO* mice resulted in a marked decrement in spatial learning and memory.

## Materials and Methods

### Mice

The generation and functional characterization of *BAF170* ^fl/fl^, [15], Emx1-Cre [17], hGFAP-Cre [18], and Nestin-CreER [19] mice has been described in earlier publications. All mutant lines were maintained on a C57BL6/J background.

### Antibodies

Polyclonal (pAb) and monoclonal (mAb) antibodies used in this study (working dilution; sources): BAF170 rabbit pAb (1:100; cat. IHC-00213 for IHC, Bethyl), Cre rabbit pAb (1:200, Millipore), Cre mouse mAb (1:100, Sigma), Tbr2 rabbit pAb (1:300; Chemicon), BrdU mouse mAb (1:40; CalTag), BrdU rat pAb (1:100; Abcam), Casp-3 rabbit pAb (1:100; Cell Signaling), Brm mouse mAb (1:50; BD Biosciences), Brg1 mouse mAb (1:50; Santa Cruz), GFAP chick pAb (1:200; Abcam), GFAP mouse mAb (1:100; Chemicon), Sox2 rabbit pAb (1:100; Chemicon), Sox2 mouse mAb (1:50; R&D Systems), Sox2 goat pAb (1:200; Santa Cruz), S100β rabbit pAb (200; Dako), Mcm2 mouse mAb (1:100; BD Biosciences), Dcx guinea pig pAb (1:200; Chemicon), NeuN mouse mAb (1:100; Chemicon), Cre mouse mAb (1:100; Sigma), Nestin mouse mAb (1:100; Chemicon), Ki67 rabbit pAb (1:200; Vector Laboratories), GS mouse mAb (1:200; Chemicon), TLX [20], Alexa 488, Alexa 568, Alexa 594, Alexa 647 (1:400; Molecular Probes).

### IHC, Quantification, and Statistical Analysis

IHC were performed as previously described [21]. Briefly, brains from young and adult mice were perfused with 4 % PFA and processed with 30 % sucrose overnight. Matched section from both wild-type and mutant brain sections were stained for antibodies for specific markers. Images were acquired with standard Axio Imager M2 (Zeiss, Oberkochen, Germany), Leica DM 6000 (Leica, Wetzlar, Germany) and confocal (Leica TCS SP5) fluorescence microscopes. Images were further analyzed with Adobe Photoshop and NeuroLucida/StereoInvestigator.

To compare the total number of cells in DG (Fig. S2d), every third coronal section (16 μm of thickness) within the DG was selected from rostral to caudal direction and stained with DAPI. Marker-positive cells within dentate gyrus (DG) of hippocampus were counted for comparison. In most cases, cell counts of six matched sections in medial DG were averaged from three biological replicates (control/cKO DG pairs). Number of lineage marker was quantified using total marker-positive cells per DG area, normalized to total number of DAPI+ cells using the following equation: Normalized number = marker-positive cell number/DAPI-positive cell number. The normalized number from control experiment then is calculated as 100 %. Statistical analyses were done using Student’s *t* test for the histological data. All bar graphs are plotted as mean ± SEM. Details of statistical analysis for histological experiment are presented in Electronic Supplementary Material (ESM) Table S1.

In behavioral experiments, the data given in figures and text are expressed as mean ± SEM. Between-group comparisons were made by either one-way analysis of variance (ANOVA) with repeated measures or *t* tests for independent samples.

*P* values given are two-tailed and considered to be significant if *p* values lower than *α* = 0.05 were obtained.

### qRT-PCR Analysis

qRT-PCR was performed as described previously [22]. Briefly, total RNA isolated from the cortex was quantified spectrophotometrically and used in quantitative RT-PCR analyses employing the QuantiTectRev. Transcription and the QuantiTect SYBR Green PCR Kits (Qiagen, Hilden, Germany). The assays were performed in triplicate and normalized to internal 18S.

### BrdU/IdU/CIdU and Tamoxifen Treatment

Dividing cells in vivo were labeled by intraperitoneal injection of BrdU (50 mg per kg of body weight)/IdU (57.5 mg per kg of body weight)/CIdU (42.5 mg per kg of body weight) in PBS for the specified durations.

Tamoxifen (Sigma) was dissolved through bursts of sonication in corn oil with a final stock concentration of 20 mg/ml. Mice were injected intraperitoneally once daily with 4 mg of tamoxifen per 20 g of body weight or sterile corn oil (vehicle) for five consecutive days.

### Behavioral Testing

For behavioral analysis mice were housed with four to five per cage in a room with a 12-h light–dark cycle (lights on at 09:00 am) with *ad libitum* access to food and water. Experiments started at the age of 3 months. The order of behavioral testing was as follows: open field, rotarod, and Morris water maze, with an inter-test interval of 1–2 days. Behavioral tests were conducted during the light phase of the day (between 10:00am to 5:00 pm), by an experimenter unaware of the genotype. All experiments were performed with permission of the Bezirksregierung Braunschweig (local Animal Care and Use Committee) in accordance with the German Animal Protection Law. Behavioral tests were performed as described previously [23]. For details, see supplemental experimental procedures.

## Results

### Expression of BAF170 in Adult Hippocampus

**Fig. 1:**
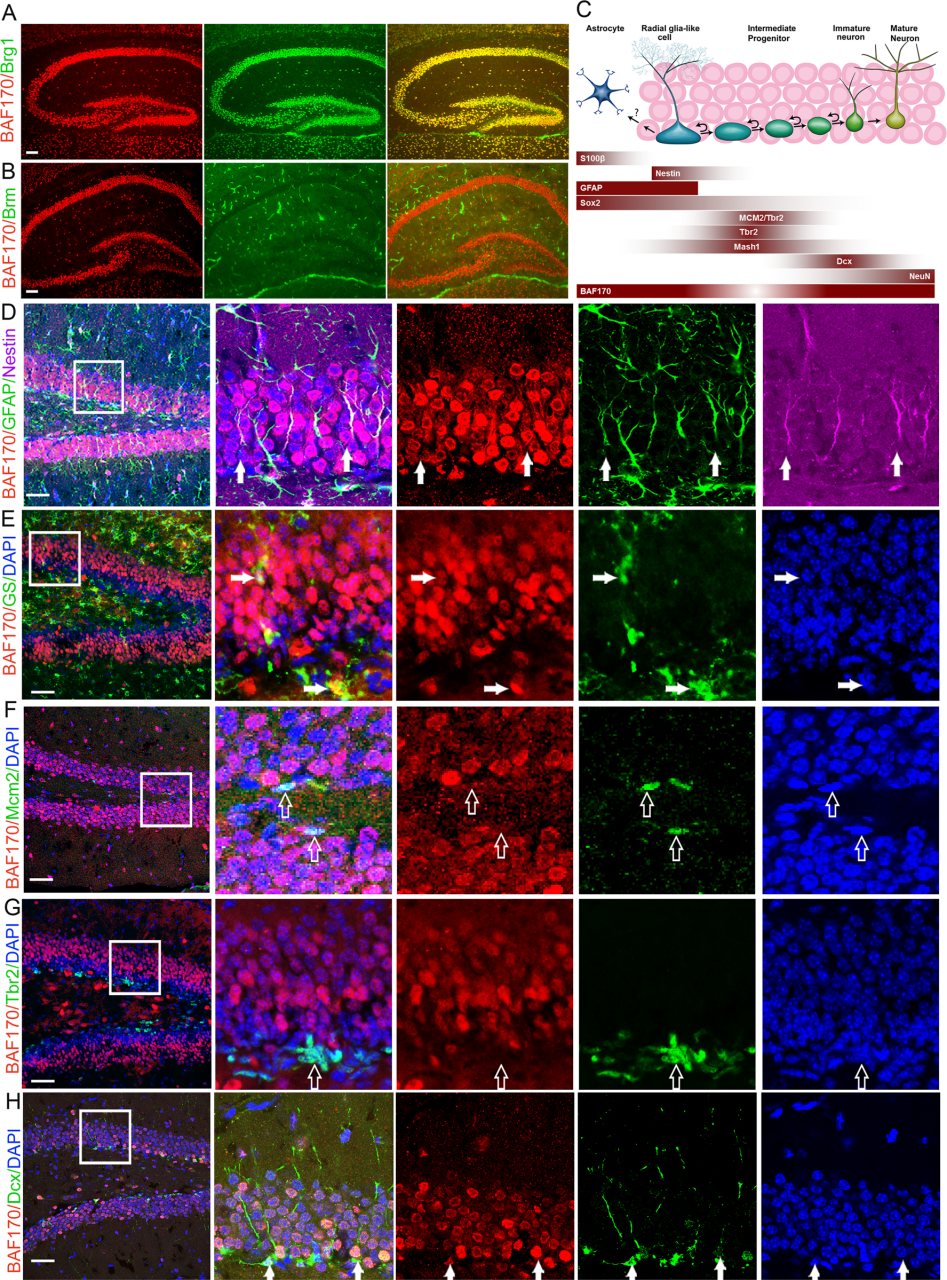
BAF170 expression in DGs of postnatal brain. **a**–**b** Images show double immunostaining of wild-type (WT) adult (4 months old) brain sections with antibodies for BAF170 (*red*) and either core subunit Brg1 or Brm (*green*). Note the full co-localization of BAF170 and Brg1 immunosignal in the adult brain hippocampus, where Brm is not expressed. **c** Schema shows a summary of BAF170 expression with different markers during adult hippocampal neurogenesis and astrogenesis. **d** Images show triple IHC analysis of DG in adult (4 months old) WT mice with antibodies for BAF170 (*red*), GFAP (*green*), Nestin (*magenta*). The *panel on the right side* shows images at a higher magnification. Overlay images of BAF170/GFAP/Nestin indicate that in SGL of DG, BAF170 is expressed in RGL progenitors (GFAP+, Nestin+, *arrows*). **e–h** Double or triple IHC analyses with BAF170 antibody (*in red*) and antibodies specifically labeling, astrocytes (GS, in **e**), neuronal progenitors (Mcm2, Tbr2 in **f/g**, see also Mash1-IHC in Fig. Fig. S1), immature neurons (Dcx, *green in* ***h***) and mature neurons (NeuN, in Fig. Fig. S1) indicated that in DG, BAF170 is absent in neuronal progenitors (Mcm2+, Tbr2+, Mash1+, *empty arrows* in **f/g**, in Fig. Fig. S1), but it is expressed astrocytes (GS+, *arrows* in **e**), neurons (Dcx+, NeuN, *arrows* in **h**, in Fig. Fig. S1). *Scale bars* = 20 μm

During the period of early neurogenesis (embryonic days E10.5–E14.5), BAF170 and Brm are transiently expressed in the cortical progenitors (ESM Fig. S1a) [15]. BAF170 recruits Brm-based BAF complex to the promoters of a set of Pax6 target genes, including those involved in the specification of late-born neuronal subtypes [15]. In adult hippocampus, double immunohistochemical (IHC) analysis with antibodies for BAF170, Brm, and Brg1 revealed almost a complete overlap of BAF170 with Brg1 staining, while expression of Brm was undetectable (Fig. 1a, b). Thus, in contrast to the cortical neurogenesis in embryonic brain where BAF170 is a component of a Brm-dependent BAF complex, in the adult hippocampus, BAF170 is possibly incorporated into a Brg1-based BAF complex as recently shown also for the adult SVZ niche [24]. The integration of BAF170 into a Brg1-dependent BAF complex in adult hippocampus was confirmed by WB analysis of BAF170 immunoprecipitates of lysates from hippocampal tissue from 2-months-old mice (ESM Fig. S1b).

During the course of adult neurogenesis in DG, upon exiting their quiescent state, radial glia-like (RGL or type 1) progenitors rapidly undergo a series of cell divisions to produce progeny with proliferative ability, including intermediate progenitors (IPs or transit-amplifying progenitors or type 2 cells) that differentiate into neuroblasts (immature neurons, or type 3 cells) [25–28].

In order to define the population of cells that express BAF170 in the DG of adult mouse brains, we performed double or triple IHC with antibodies for BAF170 and cell-type-specific markers on brain sections from young) (1 month old) and adult (4 months old) mice. The results indicated that in adult hippocampus BAF170 is expressed incell types that are involved in sequential steps of adult neurogenesis (Fig. 1c), including RGL cells (GFAP^+^/Nestin^+^ Fig. 1d), in mature astrocytes (GS^+^ cells, Fig. 1e), immature neurons (DCX^+^, Fig. 1f), mature neurons (NeuN^+^, ESM Fig. S1c). However, BAF170 expression in MCM2^+^ or Mash1^+^ or Tbr2^+^ cells, considered as IPs, was not detectable (Fig. 1g/h and ESM Fig. S1c).

### Ablation of BAF170 in Developing and Postnatal Hi Causes Abnormal Location and Proliferation of RGL Cells in SGL of DG

The hippocampus develops in the mediodorsal part of the pallium. The main features of specification of the hippocampal fields CA1, CA2, CA3 are already established during E10.5–E12.5 [29] through expression of molecular determinants in the progenitors. The Hi neurogenesis (E10.5–E18.5) is actually overlapping with field differentiation which starts at E14.5 and extends postnatally [30, 31]. Thus, starting at E14.5 until the end of corticogenesis (E18.5), RGL cells from the dortsomedial VZ generate Hi pyramidal neurons [32], while a portion of RGL cells migrate medially to differentiate into DG during the first postnatal week [33–35].

To investigate the role of BAF170, we directed Cre-dependent disruption of *BAF170* in telencephalic RGCs of the astrocyte lineage. Mice carrying the *BAF170fl/fl* allele [15] were crossed to *hGFAP-Cre* mice expressing Cre recombinase under the human glial fibrillary acidic protein promoter (*hGFAP-Cre*) [18, 36, 37] to generate *BAF170cKO_hGFAP-Cre* mutant line (*BAF170fl/fl; hGFAPCre/+)*. Double IHC with Cre and BAF170 antibodies on E14.5 cortical sections indicated that the *hGFAP* promoter drived Cre expression in most of the cortical progenitors in VZ of dorsomedial telencephalon (ESM Fig. S2a) which is in accordance with previous reports showing that although initiated at E13.5, the Cre-recombinase activity fully spreads in progenitors of mediodorsal pallium only at stage E16.5 [18, 35, 38]. Noteworthy, at E14.5 the expression of BAF170 at the ventricular surface was preserved in the mutant cortex (ESM Fig. S2a, red). Even at E18.5, the expression level of BAF170 in *BAF170cKO* cortices was not substantially different as compared with controls suggesting that the incorporated BAF170 protein in the BAF complex is stable for days (ESM Fig. S2b). In contrast, a complete loss of BAF170 expression was observed in most cortical and hippocampus cells in the brain of newborn (P1) and 4-months-old *BAF170cKO_hGFAP-Cre* mice (ESM Fig. S2c, d). Our previous data have revealed that BAF170 expression exerts an interesting dynamic during corticogenesis: a strong expression in dividing apical RGPs during early (E10.5–E14.5) neurogenesis, followed by abruptly diminished, almost undetectable expression until E17.5, andreappearing of the expression at the latest stages (E17.5–E18.5) in VZ progenitors of developing cortex as well as in the niches of adult brain neurogenesis, the forebrain SVZ and the SGZ of DG [15]. During development, the cells that form the DG are generated from distinct matrices. These include the primary matrix (VZ, SVZ of dentate primordium) formed at E13.5 in mouse, the secondary germinative matrix (the developing dentate pole) at E15.5, the tertiary matrix (in the dentate hilus) formed at prenatal ages, and the final germinative matrix established in SGZ during the second postnatal week [33]. Thus, given the dynamic of BAF170 expression pattern as mentioned above, ablation of BAF170 function in the DG via *hGFAP-Cre* line would mostly affect the late prenatal generation of the germinative matrix in the hilus and SGZ of DG in the postnatal/adult brain. Noteworthy, compared to controls, the size of both Hi proper and DG in 4-month-old *BAF170cKO_hGFAP-Cre* mice were smaller (ESM Fig. S2d). Total number of cells (DAPI+) in DG in mutants is less than that of control (ESM Fig. S2d). Moreover, IHC with Ctip2 antibody as a marker for pyramidal neurons [39] indicated that the loss of BAF170 leads to diminishing the number of Ctip2^+^ neurons in DG and CA1-2 fields in mutant brain (ESM Fig. S2e). Because mutant DG is smaller than that of control, therefore all statistical analyses thereafter were normalized to number of DAPI+ cells (ESM Table S1).

**Fig. 2:**
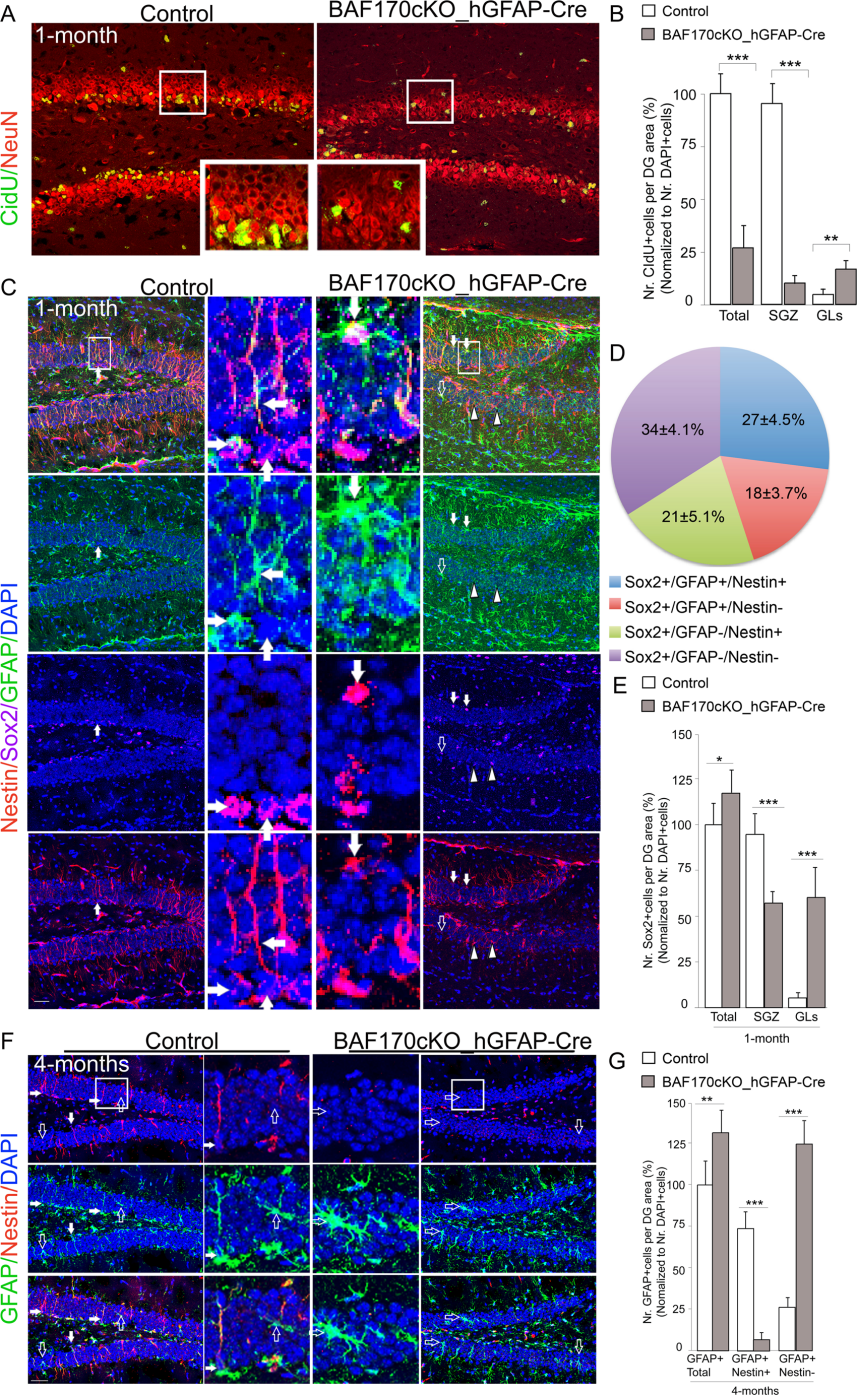
The loss of BAF170 in *BAF170cKO_hGFAP-Cre* mice affects the positioning and pool of RGL cells in DGs. **a** Double IHC analyses with NeuN antibody specifically labeling mature neurons, and CidU (injected 6 days prior analysis) indicated a severely diminished number of CidU+ cells in young (1 month) *BAF170cKO_hGFAP-Cre* mice as compared to control. **b** Diagrams show statistical analyses comparing the number of CidU+ cells (in **a**), in DGs of control and *BAF170cKo_hGFAP-Cre* mutant. **c**, **d**, **f** Images in these panels show triple (**c**) and double (**f**) IHC analysis of young (1 month old in **c**) and adult hippocampus (4 months old in **f**) with antibodies for Nestin (*red*), GFAP (*green*), and Sox2 (*magenta*). The *middle panel* in **f** shows images at a higher magnification. Mis-positioning of cells, Sox2+/GFAP+/Nestin+ cells, Sox2+/GFAP+/Nestin- cells, and Sox2+/GFAP-/Nestin+ cells, were observed in GL of DG in young (1 month old, in **c**) *BAF170cKO_hGFAP-Cre* mice (*pointed by filled*, *empty arrows*, and *head arrow*, respectively). *Filled and empty arrows* (in **f**) show GFAP+/Nestin+ cells and GFAP+/Nestin- cells, respectively. **d** The mis-positioned Sox2+ cells in GL of young *BAF170cKO*mice (shown in **c**) belong to distinct cell classes, as indicated. **e**, **g** Diagrams show statistical analyses comparing the number of Sox2+ cells (shown in **c**), RGL progenitors (Nestin+/GFAP+ cells, shown in **f**) and astrocytes (Nestin-/GFAP+ cells, shown in **f**) in DG of control and *BAF170cKO-hGFAP-Cre* mutant. Values are presented as mean ± SEM (*n* = 12). *Scale bars* = 20 μm

The granule cells of the DG are organized in an outside–in pattern: the early-generated cells migrate to the outer layers, while late-born cells located in the inner layers form the SGZ, which serves as a neurogenic niche for postnatal NSCs [3, 4]. To study proliferative capacity of NSCs in the postnatal DG, we performed a pulse labeling using thymidine CidU in control and BAF170-deficient 1-month-old animals *(*Fig. 2a). Compared to the controls, the SGZ of DG in the *BAF170cKO_hGFAP-Cre* mutant mice contained a diminished number of proliferating cells that were spread predominantly throughout the granular layer (GL) (Fig. 2a, b).

As mentioned above, the proliferating cells in SGZ of DG belong to two types: (a) GFAP^+^ and NESTIN^+^ positive slowly dividing cells (RGL cells/NSCs/ or type1 cells), and (b) amplifying progenitors (IPs, or type 2a cells), derivatives of type1 cells. Both subtypes express the SRY-related HMG (high mobility group) box transcription factor SOX2. While RGL cells slowly divide and extend GFAP+/NESTIN+ processes across the granular cell layer of DG, the type 2 cells divide more often and lack radial GFAP+/NESTIN+ processes [40–42]. To study the fate of the mis-positioned CidU+ proliferating cells in the DG of *BAF170cKO_hGFAP-Cre* mice, we performed triple IHC analysis using antibodies for Sox2, Nestin, and GFAP. In the wild type DG, most of the GFAP+ and NESTIN+ cells were RGL cells with cell bodies located in the SGZ, extending GFAP+ processes that pass through the granular cell layer of DG and protrude outward (Fig. 2c). In contrast, in the DG of *BAF170cKO_hGFAP-Cre* animals, many GFAP+ and NESTIN+ RGLs were mis-positioned in the outer layers, having randomly branching fibers of elongated radial morphology (Fig. 2c, arrows).

Immunostaining with Sox2 antibody labels the cell nucleus of both postnatal NSC and astrocytes in the DG [43, 44]. In the absence of BAF170, the number of Sox2+ cells in the mutant brain was slightly enhanced as compared with the control (Fig. 2c, e). Consistent with the results from the CidU-pulse labeling experiment,, while in the control brain, most of the Sox2+ cells were located in the SGZ, with only a few Sox2+ cells populating the granular layer (GL) (Fig. 2c, e), in the BAF170-deficient DG, almost equal numbers of Sox2+ cells were found in the SGZ and in the outer layers of the dentate blade (Fig. 2c, e, arrows). To assess the molecular identity of the mislocated Sox2+ cells after loss-of-function (LOF) of BAF170, triple IHC labeling with antibodies against Sox2, Nestin, and GFAP was performed. The results (Fig. 2c, d) indicated that 27 ± 4.5 % of the Sox2+ cells were Nestin+ and GFAP+ cells (Fig. 2c, solid arrowheads), while lower proportions of the Sox2+ cells were either GFAP+ (Fig. 2c, 18 ± 3.7 % arrowhead) or Nestin+ (Fig. 2c, 21 ± 5.1 % empty arrows). In addition, 34 ± 4.1 % of the Sox2+ cells were Nestin−/GFAP− cells. These data indicate that the mislocated Sox2+ cells are not only RGL progenitors (type 1 neural stem cells, Sox2+/GFAP+/Nestin+), but also cells at different stages of differentiation [4, 5].

### Enhanced Astrogenesis in DG of BAF170-Deficient Mice

Studies with long-term lineage tracing in vivo indicated that RGL precursors in the adult DG have the capacity to self-renew and undergo multi-lineage differentiation, including astrogenesis [43]. RGL cells in DGs express neuroepithelial stem cell marker Nestin as well as markers for immature astrocytes and radial glial progenitors such as GFAP and brain lipid-binding protein (BLBP). Compared to control, immunostaining with GFAP antibody revealed a higher abundance of GFAP+ immnunostaining signal in entire tissue section, including DG of 1-month-old *BAF170cKO_hGFAP-Cre* mice (Fig. 2c). To examine whether loss of BAF170 affects the astrogenesis, we performed triple IHC with antibodies for Nestin, GFAP, and BLBP on brain sections from 1.5-months transgenic and control mice (ESM Fig. Fig. S3). Similarly, the DG of the BAF170-deficient brains contained more GFAP+/BLBP+ cells as compared to the controls. Notably, while most of the cells in DG of the control brain extended radial fibers that were immunoreactive for Nestin, GFAP, and BLBP (ESM Fig. Fig. S3, filled arrows), most of the GFAP+/BLBP+ cells in the mutant DG were with astrocyte morphology, expressing Nestin either at a low level or lacked Nestin expression (ESM Fig. Fig. S3, empty arrows). These findings suggested that the presence of BAF170 subunit in the complex is important for maintenance of RGL cell fate and/or astrocyte differentiation in postnatal DGs.

During postnatal hippocampal astrogenesis, the expression of Nestin is down-regulated, while the expression BLBP is augmented [45–47]. To further examine whether loss of BAF170 in *BAF170cKO_hGFAP-Cre* mice affects the pool of RGL cells and astrogenesis in DGs, we performed double IHC with antibodies for Nestin and GFAP on brain sections from 4-months-old *BAF170cKO* and control mice (Fig. 2f, g). The total number of GFAP+ cells was significantly increased in the DG of the mutant as compared to the control brains. In addition, in the DG of control mice, most of the GFAP+ cells with long radial fibers were also positive for Nestin (filled arrows), and few GFAP+ cells with astrocytic morphology were negative for Nestin (empty arrows, Fig. 2f, g). Strikingly, in the BAF170-deficient DG, the Nestin+ cells with long radial fiber morphology in SGZ were largely lost indicating that the pool of RGL cells for adult neurogenesis was substantially diminished in the mutant DG.

Conditional deletion of BAF170 in embryonic cortex starting at the onset of neurogenesis via Emx1-Cre line strongly affects the neurogenesis; however, apoptosis in this structure has not been detected, neither during development nor at postnatal stages (P10) [15]. Likewise, we did not observe apoptosis in hippocampus with the deletion of BAF170 at mid-gastration in *BAF170KO_hGFAPCre* mutants as indicated by Casp3 IHC analysis at stages of E18.5 and 1.5 months old (ESM Fig. S2f). Therefore, the observed reduction of the RGL cell pool in the loss of BAF170 in DGs in *BAF170KO_hGFAPCre* mice could not be based on cell death, but rather could involve a depletion of the RGL cell pool. In a further support, we found that compared to controls, the expression of TLX, an important regulator of the maintenance and self-renewal of postnatal/adult NSCs, [7, 48], was substantially declined in the DG of 2.5-month-old *BAF170cKO* animals. Quantitative estimation revealed significantly reduced numbers of TLX+ cells in DG in a lack of BAF170 as compared to control (ESM Fig. S4).

**Fig. 3:**
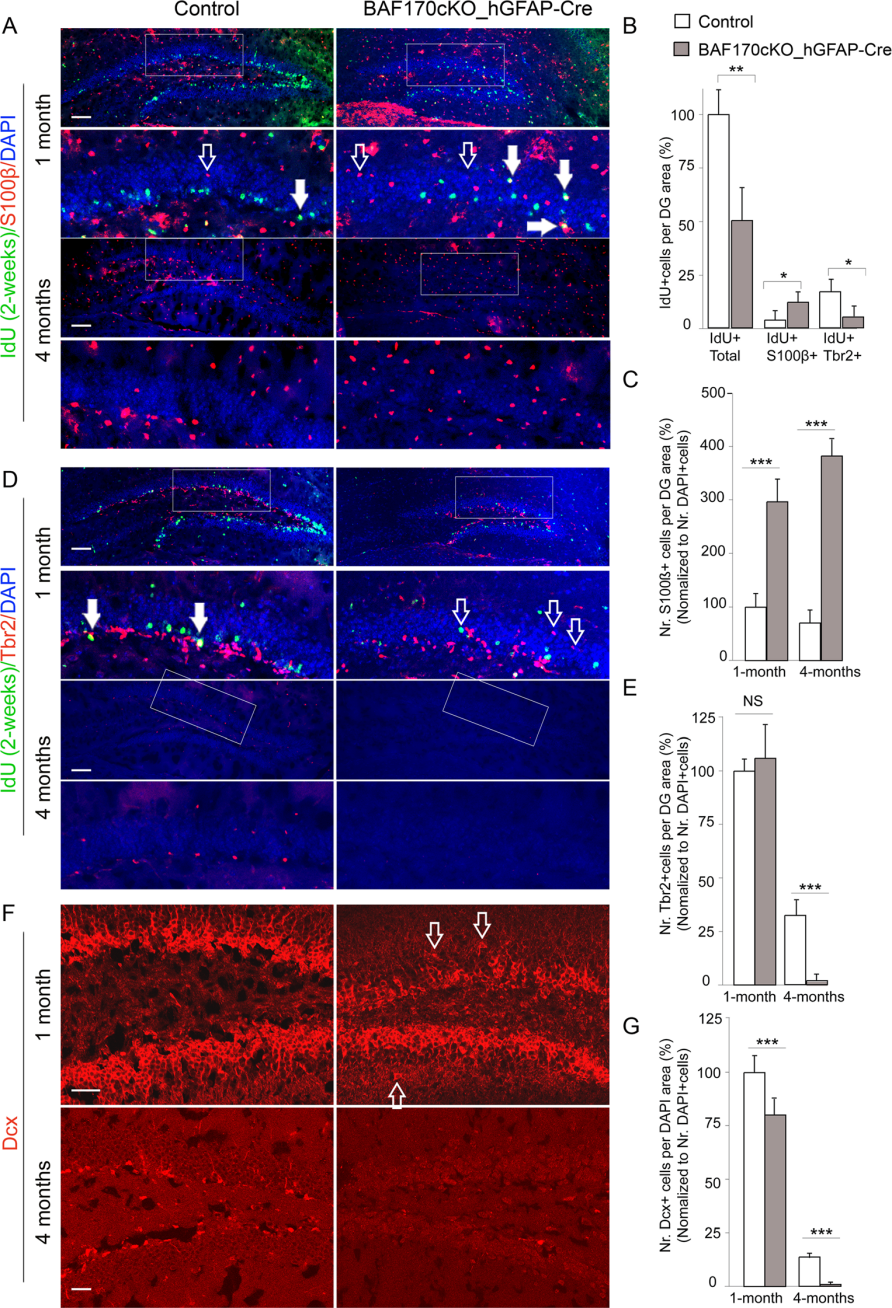
Loss of BAF170 during lates stages and postnatal neurogenesis leads to increase of the terminal astrocytic differentiation at the expense of neurogenesis in DG. **a**, **d**, **f** Images show double (**a**, **d**) or single (**f**) IHC analyses of DG tissues of young (1 month old; IdU injection for 2 weeks prior analysis) and old (4 months old) mice with antibodies specifically labeling astrocyte (S100β), neuronal IPs (Tbr2), immature neurons (Dcx). *Filled arrows* show S100β+/IdU+ cells (in **a**) and Tbr2+/IdU+ cells (in **d**). *Emptry arrows* point mis-locations of S100β+ cells (in **a**), Tbr2+ cells (in **d**), and Dcx+ cells (in **f**) in GL of young (1 month old) *BAF170cKO_hGFAP-Cre* mice. **b** The diagrams represent results from statistical analyses, comparing the number of total IdU+ cells, IdU+/S100β+ astrocytes (in the selected areas in **a**) and IdU+/Tbr2+ IPs (in the selected areas in **d**) in DGs of 1 month old *BAF170cKO_hGFAP-Cre* mutants and control. **c**, **e**, **g** Diagrams show statistical analysis data comparing the number of S100β+ astrocytes (in the selected areas in **a**), Tbr2+ neuronal IP progenitors (in the selected areas in **d**) and Dcx+ immature neurons (in the selected areas in **f**) in control and BAF170 mutant DGs at indicated ages. Values are presented as mean ± SEM (*n* = 12). *Scale bars* = 20 μm

To examine further whether BAF170-deficiency might influences the cell type differentiation from mislocated RGL progenitors, we performed IHC analysis with antibodies labeling IPs (Tbr2), astrocytes (S100β), and immature neurons (Dcx) on brain sections from young control and *BAF170cKO* mice. In GLs of the BAF170 mutant, we observed Tbr2+ IPs, S100β+ astrocytes, and Dcx+ immature neurons (Fig. 3, empty arrows), suggesting that the mis-positioned RGLs in DG of young *BAF170cKO* mice might have lost their self-renewal/proliferative abilities and possibly undergo a faster differentiation in the ectopic location, compared to those in the control brain. Remarkably, the DG in *BAF170cKO_hGFAP-Cre* mice exhibited a profound increase of the numbers of GFAP+/Nestin− astrocytes (empty arrows) as compared to the control mice (Fig. 2f, g). Together, these findings suggest that elimination of BAF170 during late and postnatal cortical neurogenesis substantially diminishes the pool of NSC in SGZ of DG, the RGL cells show ectopic distribution across the GLs of DG and possibly undergo a premature differentiation predominantly into mature astrocytes.

To evaluate the cell fate decisions in postnatal DG upon BAF170 elimination, we injected 2-week-old mice with IdU and analyzed the phenotype 2-weeks later (Fig. 3). The analysis indicated that loss of BAF170 led to diminishing the number of IdU+ cells in the DG of mutant as compared to the control mice (Fig. 3a, b). Co-labeling of IdU+ cells with the astrocytic marker S100β indicated a significant increase of IdU+/S100β+ cells in the DG of *BAF170cKO* mice as compared to the controls (Fig. 3a, b). The enhanced astrogenesis was confirmed by a significantly greater number of mature astrocytes (S100β+ or GS+ cells) detected in the DGs of young and adult *BAF170cKO* ascompared with wild-type mice (Fig. 3a–c, ESM Fig. S5B/D). These findings indicate that RGL cells indeed precociously differentiate into astrocytes in the DG of *BAF170cKO-hGFAP-Cre* mice.

Next, we examined whether differentiation of RGL cells into neuronal lineage was also affected. Co-immunostaining with antibodies for IdU and Tbr2, a specific marker for IPs [49], revealed a decreased number of double IdU+/Tbr2+ cells in DGs of *BAF170_hGFAP-Cre*, compared to the control mice, suggesting that there is either reduced production of IPs and/or decreased rate of IP proliferation. We then examined the expression of IP marker Mcm2, which is active throughout proliferative cell cycle, but becomes inactive in quiescent and differentiated cells [50]. The results revealed that the loss of BAF170 led to a significantly lower number of proliferating cells in the DG of young *BAF170cKO* mice as compared to that of control mice (ESM Fig. S5a, c). In addition, IHC with antibody for Dcx, a marker of immature neurons, indicated that the DG of 1-month-old *BAF170_hGFAP-Cre* mice contained a diminished number of Dcx+ cells as compared to the control (Fig. 3f, g). Remarkably, while Dcx+ cells, Tbr2+ cells, and Mcm2+ cells were generated in the DG of 4-month-old wild-type brains, these cells were rarely detectable in the DG of *BAF170_hGFAP-Cre* mice (Fig. 3f, g, ESM Fig. S5a, c). Taken together, these findings suggest that in absence of BAF170 during late and postnatal hippocampal neurogenesis RGL cells in DG differentiate preferably into astrocytes rather than generating neuronal progenitors and neurons.

### Ablation of BAF170 During Adult Brain Neurogenesis

**Fig. 4:**
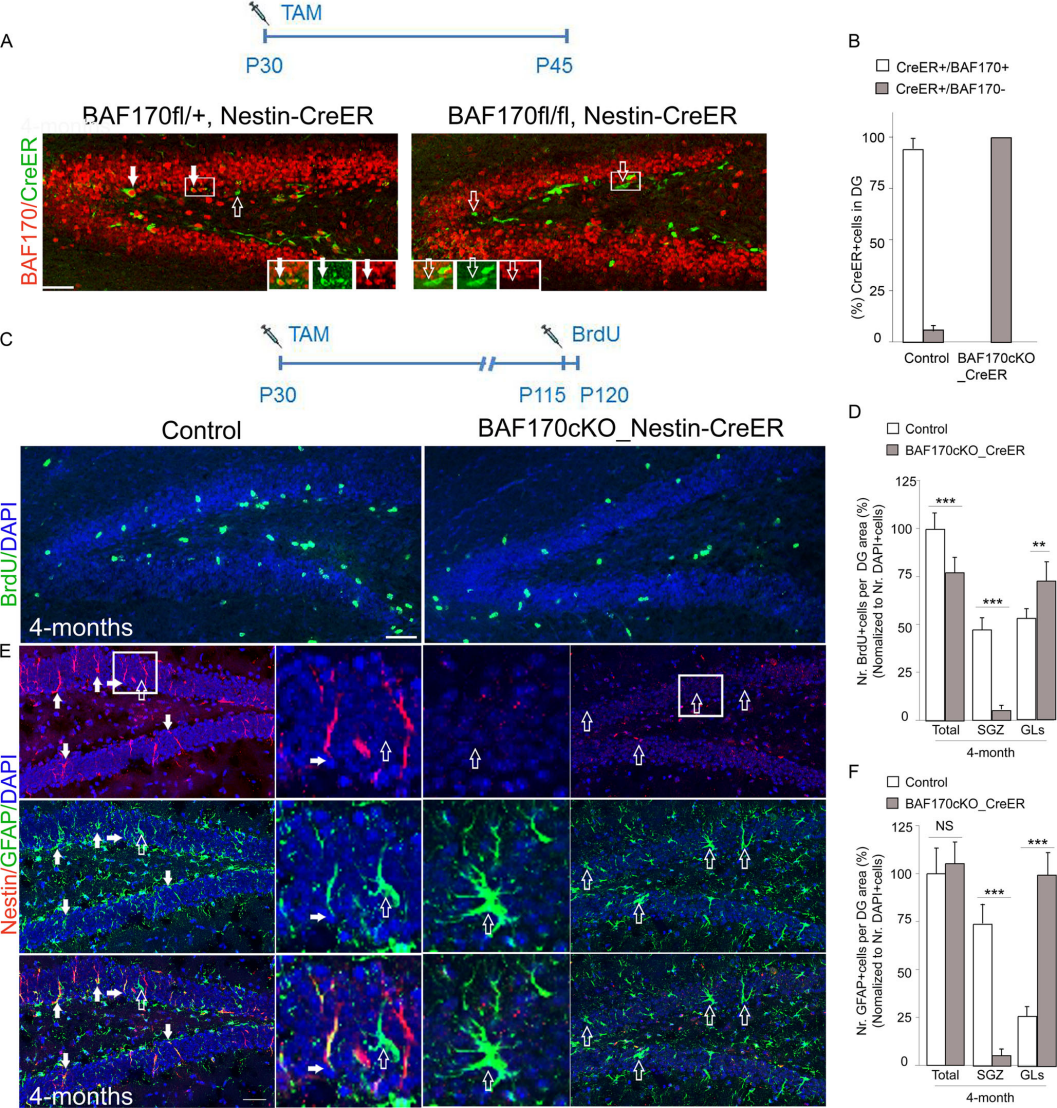
Affected adult neurogenesis in DG in inducible *BAF170cKO_Nestin-CreER* mutant. **a**–**b** Representative images of the control and *BAF170c*KO_*Nestin*-CreER DGs with a schema of the tamoxifen (TAM) treatment. **a** 15 days after the TAM injection, most of the CreER+ cells are immunopositive with BAF170 antibody (*filled arrows*) in DG of control animals (*BAF170fl/+; Nestin-CreER)*, but the BAF170 protein was fully lost in DG of in *BAF170c*KO_*Nestin*-CreER mice (*empty arrows*). **b** The histogram illustrates the proportion of CreER+/BAF170+ cells and CreER+/BAF170− cells out the total number of CreER+ cells in DG of adult mutant and control animals. **c** The schema shows time points of TAM, BrdU treatment, and harvesting of brain tissues. Images of IHC analysis with BrdU antibody (*green*) indicate a loss of BrdU+ cells in the subgranular zone (SGZ) of 4-months-old *BAF170c*KO_*Nestin*-CreER mice as compared to control. **d** The histogram presents the proportion of BrdU+ cells in SGZ and granular layer (GL) in control and *BAF170c*KO_*Nestin*-CreER mice. **e** Images show double IHC staining with antibodies for Nestin, GFAP. The *middle panel* shows images at a higher magnification. *Filled and empty arrows* point GFAP+/Nestin+ cells and GFAP+/Nestin− cells, respectively. **f** The histogram shows statistical analyses comparing the number of RGL progenitors (Nestin+/GFAP+ cells, *filled arrows* in **e**) and astrocytes (Nestin−/GFAP+ cells, *empty arrows* in **e**) in DG of control and *BAF170c*KO_*Nestin*-CreER mutant mice. Values are presented as mean ± SEM (*n* = 12). *Scale bars* = 20 μm

To evaluate the role of BAF170 in adult brain neurogenesis in a more restricted manner, next we deleted the BAF170 in SGZ of DG in adult *BAF170* ^*fl/fl*^ mice (4 months old) applying conditional inactivation of BAF170 after tamoxifen (TAM) administration to activate Cre recombinase. We crossed *BAF170* ^*fl/fl*^mice with a mouse line that contains a TAM-inducible Cre transgene under the control of the *Nestin* promoter (*Nestin*–CreER) [19] to generate a *BAF170*cKO_*Nestin*-CreER mouse line. Such 1-month-old double transgenic mice were injected with TAM (Fig. 4a, b). Two weeks thereafter, the expression of BAF170 was completely abolished in the CreER+ cells in DG of the mutants, confirming efficient Cre-mediated deletion (Fig. 4a).

To study the proliferative ability of the NSC in the SGZ, we performed a BrdU pulse labeling for 5 days of 4-months-old control and *BAF170c*KO_*Nestin*-CreER mice (Fig. 4c, d). Similarly to *BAF170*cKO_*hGFAP*-Cre mice (Fig. 2a, b), the IHC analysis with BrdU antibody revealed that compared to the control, the mutant DG contained a significantly lower number of proliferating BrdU+ cells (Fig. 4c, d). In addition, most of the BrdU+ cells in the mutant DG were distributed throughout the GL rather being located in SGZ (Fig. 4c, d), suggesting that RGL cells are mislocated upon eliminantion of BAF170 function in SGZ of the adult DG. To further investigate whether the loss of BAF170 in adult *BAF170*cKO_*Nestin*-CreER mice influences the pool of RGL cells, we examined the expression of two RGL cell markers, GFAP and Nestin (Fig. 4e, f). The total number of GFAP+ cells in the mutant DGs was not significant difference compared to that of control. In accordance with the detected loss of adult NSC in DGs of *BAF170_hGFAP-Cre* mutants, also upon postnatal and restricted to SGZ deletion of BAF170, the number of GFAP+/Nestin+ RGL cells (filled arrows) was largely lost in the mutant DGs compared to the control. Moreover, reminiscent to the *BAF170cKO_hGFAP* mutant brain, we also found in DGs of adult *BAF170*cKO_*Nestin*-CreER mice an increased numbers of GFAP+/Nestin− astrocytes cells (empty arrows) compared to the control, indicated that in vivo ablation of BAF170 during late corticogenesis as well as specifically in SGZ of adult brain affect generation/maintenance of RGL progenitors in DG which seem to prematurely differentiate into mature astrocytes (Figs. 2 and 4 *)*.

### Impaired Accuracy of Hippocampus-Dependent Place Responding in *BAF170cKO_hGFAP-Cre* Mice

Analysis of behavioral phenotypes of 3-month-old *BAF170cKO_hGFAP-Cre* mutant mice and their control littermates showed no significant group differences on the rotarod (main effect of days: *F*[1, 20] = 7.553, *p* = 0.012; genotype × days interaction: *F*[1, 20] = 0.080, *p* = 0.780; main effect of genotype: *F*[1, 20] = 0.509, *p* = 0.484, repeated measures ANOVA; ESM Fig. S6a) and open-field test (time spent in zones: periphery: T[20] = 0.729, *p* = 0.475; intermediate: T[20] = 0.082, *p* = 0.935; center: T[20] = 1.056, *p* = 0.303; total distance moved: T[20] = 0.697, *p* = 0.494, *t* test for independent samples; ESM Fig. S6b, c). Thus, motor coordination and exploratory activity in a novel environment had not been affected by *BAF170cKO-hGFAP-Cre* deficiency.

**Fig. 5:**
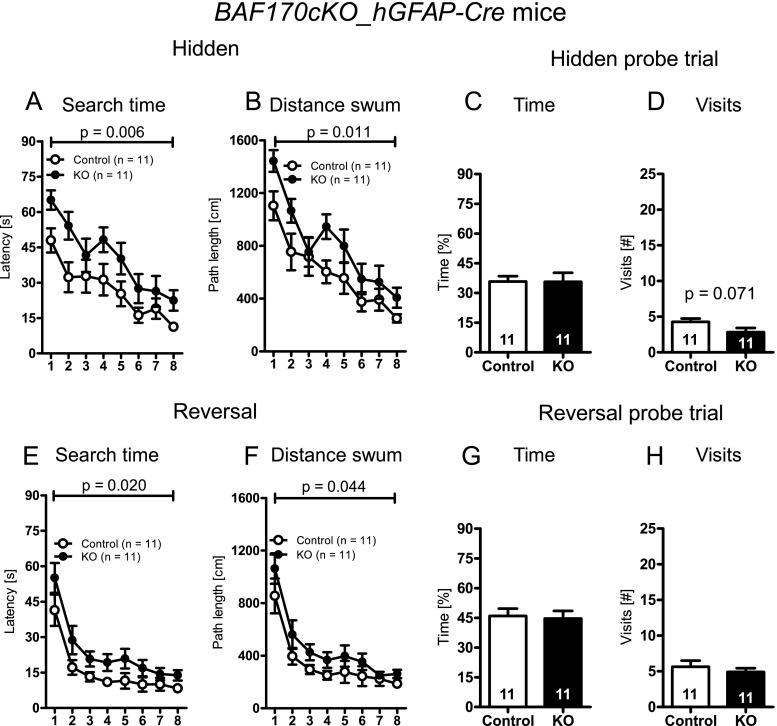
The accuracy of place responding is altered in adult *BAF170cKO-hGFAP-Cre* mice. *BAF170cKO-hGFAP-Cre* mice showed unaltered visible platform task performance. Performance of *BAF170cKO-hGFAP-Cre* mice in the hidden platform task, in terms of (**a**) escape latency and (**b**) distance swum until the platform was reached was significantly higher in the mutant as compared to control mice (*p* values refer to significant main effects of genotype obtained with an ANOVA with repeated measures). Although control and *BAF170cKO-hGFAP-Cre*mice spent similar time in the target quadrant during the hidden platform probe trial (**c**), *BAF170cKO-hGFAP-Cre* mice showed a significant decrease in the number of former platform position crossings (**d**); *p* value refers to a *t* test for independent samples. *BAF170cKO-hGFAP-Cre* mice showed significantly higher search times (**e**) and distance swum (**f**) to locate the platform during the reversal learning test (*p* values refer to significant main effects of genotype, ANOVA with repeated measures). Reversal probe trial performance was similar between mutant and control mice both in terms of the time spent in the former platform quadrant as well as in terms of former platform position crossings (**g**, **h**). Data is presented as mean ± SEM. *Numbers given in the bars* refer to corresponding sample sizes

In order to assess spatial learning and memory capacities as well as reversal learning performance, we tested BAF170-deficient and control mice in the Morris water maze test. In the visible platform task (Hi-independent stage of the test), *BAF170cKO_hGFAP-Cre* mutants performed similar to control animals suggesting intact vision, swimming ability, and motivation to escape from the pool (search time: main effect of days: *F*[1, 20] = 21.259, *p* < 0.001; genotype × days interaction: *F*[1, 20] = 0.637, *p* = 0.434; main effect of genotype: *F*[1, 20] = 0.011, *p* = 0.919; distance swum: main effect of days: *F*[1, 20] = 18.765, *p* < 0.001; genotype × days interaction: *F*[1, 20] = 0.713, *p* = 0.409; main effect of genotype: *F*[1, 20] = 0.565, *p* = 0.461, repeated measures ANOVA; data not shown). Across the days of hidden platform acquisition (Hi-dependent stage of the test) the mice, irrespective of genotype, showed a significant reduction in escape latencies and distance swum to reach the hidden platform (main effect of days, escape latency: *F*[7,140] = 14.460, *p* < 0.001; distance swum: *F*[7,140] = 20.295, *p* < 0.001, repeated measures ANOVA; Fig. 5a, b). However, compared to controls, the *BAF170cKO_hGFAP-Cre* mice exhibited significantly higher escape latencies to reach the hidden platform (main effect of genotype: *F*[1, 20] = 9.385, *p* = 0.006, repeated measures ANOVA; Fig. 5a). Likewise, the distance swum, until the platform was located, was significantly higher in the *BAF170cKO_hGFAP* as compared to the control mice (main effect of genotype: *F*[1, 20] = 7.915, *p* = 0.011, repeated measures ANOVA; Fig. 5b). However, no significant genotype × days interaction was found. The deficit of the BAF170-deficient mice was already evident on the first day of hidden platform training (escape latency, *BAF170cKO_hGFAP-Cre* 65.06 ± 13.69 vs. control 47.94 ± 17.06, T[20] = 2.60, *p* = 0.017; distance swum, *BAF170cKO_hGFAPCre* 1443.58 ± 273.88 vs. control 1104.22 ± 362.92, T[20] = 2.476, *p* = 0.022, *t* test for independent samples) and persisted until the last day of training (escape latency, *BAF170cKO*_hGFAPC-Cre 22.48 ± 14.34 vs. control 11.30 ± 5.39, T[20] = 2.419, *p* = 0.025; distance swum, *BAF170cKO* 407.14 ± 251.44 vs. control 250.13 ± 104.23, T[20] = 1.913, *p* = 0.070, *t* test for independent samples). Furthermore, the escape latency and distance swum curves of the two groups appear to run in parallel arguing against differences in the speed of spatial learning. These results suggest that the differences observed in the escape latencies to the hidden platform and distance swum until the hidden platform was finally located were not due to a deceleration in the speed of spatial learning in the *BAF170cKO_hGFAP-Cre* mice. Rather it seems that the*BAF170cKO_hGFAP-Cre* mutant mice suffer from an impaired accuracy of place responding [51], possibly due to an impaired stability of hippocampal place fields leading to a blurring of the cognitive spatial map that is used for spatial navigation [52]. This idea is corroborated by the results of the hidden platform probe trial. The time spent in the former platform quadrant was not significantly different between *BAF170cKO_hGFAP-Cre* and control mice (T[20] = 0.025, *p* = 0.981; *t* test for independent samples, Fig. 5c), suggesting that the mutant mice had acquired some knowledge about the rough spatial location of the hidden platform. In contrast, the number of crossings of the former platform location was lower in the *BAF170cKO_hGFAP-Cre* mice as compared to the controls (T[20] = 1.910, *p* = 0.071; *t* test for independent samples, Fig. 5d), suggesting that they have not learned the exact spatial location of the platform position. A similar pattern of results was obtained in the reversal learning test. The mice (irrespective of genotype) significantly improved their performance across the days of reversal learning (main effect of days, escape latency: *F*[7,140] = 29.309, *p* < 0.001; distance swum: *F*[7,140] = 26.500, *p* < 0.001, repeated measures ANOVA; Fig. 5e, f). However, again, the *BAF170cKO_hGFAP-Cre* mice showed significantly higher escape latencies and swum a longer distance until they could locate the hidden platform (that had been moved to a novel location) as compared to the controls (main effect of genotype, escape latency: *F*[1, 20] = 6.440, *p* = 0.020; distance swum: *F*[1, 20] = 4.620, *p* = 0.044, repeated measures ANOVA; Fig. 5e, f). As expected, no significant genotype × days interaction was found (escape latency: *F*[7,140] = 0.475, *p* = 0.852; distance swum: *F*[7,140] = 0.347, *p* = 0.931, repeated measures ANOVA). Similar to the initial hidden platform acquisition phase (although slightly more variable), the difference between *BAF170cKO_hGFAP-Cre* and control mice was evident from the very beginning of the reversal learning test (search time, conventional *BAF170cKO* 55.05 ± 20.92 vs. control 41.38 ± 22.05, T[20] = 1.492, *p* = 0.151; distance swum, conventional *BAF170cKO* 1063.44 ± 383.54 vs. control 855.139 ± 438.21, T[20] = 1.186, *p* = 0.249, *t* test for independent samples) and also persisted until its termination (search time, *BAF170cKO_hGFAP-Cre* 13.83 ± 7.29 vs. control 8.29 ± 4.24, T[20] = 2.179, *p* = 0.041; distance swum, *BAF170cKO_hGFAP-Cre* 261.04 ± 106.31 vs. control 186.46 ± 84.95, T[20] = 1.818, *p* = 0.084, *t* test for independent samples). No significant differences between the *BAF170* mutant and control mice were found in the reversal learning probe trial (time spent in target quadrant: T[20] = 0.255, *p* = 0.801; number of crossings: T[20] = 0.729, *p* = 0.474, *t* test for independent samples; Fig. 5g, h).

In conclusion, ablation of BAF170 during late and postnatal Hi-neurogenesis appears to impair similarly both the accuracy of place responding in the hidden platform and reversal learning version of the Morris water maze test.

### Performance of TAM-Inducible *BAF170cKO_Nestin-CreER* Mice After Restricted Elimination of *BAF170* Function in SGZ of DG in Adult Brain

The hippocampus proper of *BAF170_hGFAP-Cre* mice was smaller compared with the controls (ESM Fig. S2d/f), suggesting that aberrant Hi-development during late corticogenesis and after birth could be an explanation for the impaired accuracy of place responding of BAF170_hGFAP-Cre mice detected in the Morris water maze test. Therefore, we decided to test also the TAM-inducible *BAF170cKO_NESTIN-CreER* mice at the age of 3 months in the Morris water maze test. These mutants developed normally until the age of 4 weeks when *BAF170cKO*_Nestin-CreER mice were treated with TAM to completely knock down *BAF170* until the end of the sixth postnatal week.

Similar to the *BAF170cKO_hGFAP-Cre* mice, no significant differences between *BAF170cKO_Nestin-CreER* and control mice were observed on the rotarod (main effect days: *F*[1, 12] = 0.156, *p* = 0.700; genotype × days interaction: *F*[1, 12] = 0.122, *p* = 0.733; main effect of genotype: *F*[1, 12] = 0.024, *p* = 0.880, repeated measures ANOVA; ESM Fig. S6d) and open-field test (time spent in zones: periphery: T[12] = 0.866, *p* = 0.404; intermediate: T[12] = 1.144, *p* = 0.275; center: T[12] = 0.192, *p* = 0.851; total distance moved: T[12] = 0.398, *p* = 0.698, *t* test for independent samples; ESM Fig. S6e, f). These results suggest that motor coordination and balancing performance, as well as exploration of a novel environment, has not been affected by *BAF170* deficiency that has been initiated after the fourth postnatal week.

In the Morris water maze test, no significant differences between the inducible *BAF170cKO-NESTINCre-ER* and control mice were observed during the visible platform test, indicating that vision, swimming ability, and the motivation to escape from swimming in relatively cold water was not affected by the elimination of *BAF170* at the age of 4 to 6 weeks (search time: main effect of days: *F*[1, 12] = 41.257, *p* < 0.001; genotype × days interaction: *F*[1, 12] = 0.070, *p* = 0.796; main effect of genotype: *F*[1, 12] = 0.050, *p* = 0.827; distance swum: main effect of days: *F*[1, 12] = 22.501, *p* < 0.001; genotype × days interaction: *F*[1, 12] = 0.703, *p* = 0.418; main effect of genotype: *F*[1, 12] = 0.020, *p* = 0.889, repeated measures ANOVA; data not shown).

**Fig. 6:**
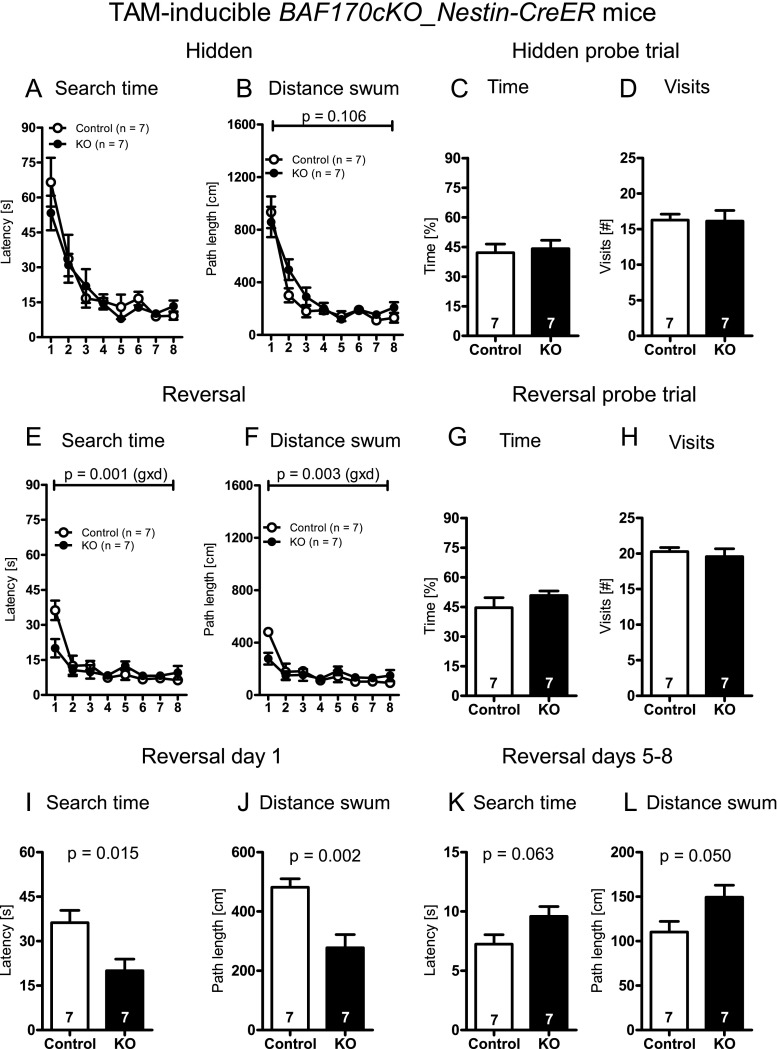
Impaired spatial and reversal learning in the Morris water maze paradigm of TAM-inducible *BAF170cKO-Nestin-CreER* mice. TAM-inducible *BAF170cKO-Nestin-CreER* mice showed no changes in hi-independent visible platform training in Morris water-maze (not shown). Escape latencies of TAM-inducible *BAF170cKO-Nestin-CreER* mice in the hidden platform task were similar to controls (**a**), but the distance swum to find the platform (**b**) was slightly increased in the TAM-inducible *BAF170cKO-Nestin-CreER* mice (*p* value refers to the main effect of genotype obtained with an ANOVA with repeated measures). No significant difference between control and TAM-inducible *BAF170cKO-Nestin-CreER* mice were observed during the hidden platform probe trial (**c**, **d**). In the reversal test a significant genotype **×** days interaction was evident in both escape latency (**e**) and distance swum (**f**) readouts (*p* values refer to significant genotype × days interactions obtained with an ANOVA with repeated measures). Reversal probe trial performance was similar between TAM-inducible *BAF170cKO-Nestin-CreER*and control mice both in terms of the time spent in the former platform quadrant as well as in terms of former platform position crossings (**g**, **h**). A more detailed analysis of the reversal learning test (**i**–**l**) revealed shorter escape latencies (**i**) and distances swum (**j**) on the first day of reversal learning, but a longer mean escape latency (**k**) and mean distance swum (**l**) across the reversal days 5 to 8 in the TAM-inducible *BAF170cKO-NestincpsCreER* mice as compared to the controls. Data is presented as mean ± SEM. *Numbers given in the bars* refer to corresponding sample sizes

During the days of the hidden platform task, the mice (independent of the genotype) displayed a significant reduction in the escape latencies and the distance swum to reach the hidden platform (main effect of days, escape latency: *F*[7, 53] = 24.443, *p* < 0.001; distance swum: *F*[7, 53] = 39.295, *p* < 0.001, repeated measures ANOVA; Fig. 6a, b). While the escape latency was not significantly different between the inducible *BAF170cKO* and control mice (main effect of genotype: *F*[1, 12] = 0.327, *p* = 0.578, repeated measures ANOVA; Fig. 6a), there was a trend for an increase in the distance swum to reach the hidden platform in the inducible *BAF170cKO_NESTIN-creER* mice (main effect of genotype: *F*[1, 12] = 3.052, *p* = 0.106, repeated measures ANOVA; Fig. 6b), suggesting a moderate impairment in spatial learning. No significant difference between the genotypes was found neither for the first (search time, *BAF170cKO*: 52.86 ± 19.64 vs. control: 65.86 ± 27.78, T[12] = 1.010, *p* = 0.332; distance swum, *BAF170cKO*: 858.29 ± 304.64 vs. control: 932.86 ± 318.52, T[12] = 0.448, *p* = 0.662, *t* test for independent samples) or the last day (search time, *BAF170cKO*: 12.71 ± 6.42 vs. control: 8.71 ± 4.89, T[12] = 1.311, *p* = 0.214; distance swum, *BAF170cKO*: 208.00 ± 107.64 vs. control: 130.14 ± 96.675, T[12] = 1.424, *p* = 0.180, *t* test for independent samples) of the hidden platform training. Similarly, no significant genotype × days interaction was evident (escape latency: *F*[7, 53] = 0.697, *p* = 0.674; distance swum: *F*[7, 53] = 1.045, *p* = 0.406, repeated measures ANOVA; Fig. 6a, b). Hidden platform probe trial performance was also comparable between the two groups (time spent in target quadrant: T[12] = 0.335, *p* = 0.744; number of crossings: T[12] = 0.083, *p* = 0.935, *t* test for independent samples; Fig. 6c, d). In sum, after normal embryonic and early postnatal development, the TAT-inducible knockout of *BAF170* in SGL of DG in the mouse at the age of 4 weeks, leads to only minor spatial learning deficits in the hidden platform version of the Morris water maze test.

During the days of the reversal learning test, the mice (independent of the genotype) showed a significant reduction in the escape latencies and the distance swum to reach the hidden platform (main effect of days, escape latency: *F*[7, 53] = 19.104, *p* < 0.001; distance swum: *F*[7, 53] = 15.343, *p* < 0.001, repeated measures ANOVA; Fig. 6e, f). While there was no significant main effect of genotype on escape latencies or the distance swum to reach the hidden platform (escape latency: *F*[1, 12] = 0.582, *p* = 0.460; distance swum: *F*[1, 12] = 0.320, *p* = 0.582, repeated measures ANOVA; Fig. 6e, f), we found significant genotype × days interactions for the readouts escape latency and distance swum (escape latency: *F*[7, 53] = 4.046, *p* = 0.001; distance swum: *F*[7, 53] = 3.428, *p* = 0.003, repeated measures ANOVA; Fig. 6e, f), indicating the existence of between-group differences regarding the course of reversal learning. A more detailed analysis of the reversal learning course of the two groups revealed that the inducible *BAF170cKO* mice showed a significantly lower escape latency and a reduced swimming distance on the first day of reversal learning (escape latency: T[12] = 2.834, *p* = 0.015, distance swum: T[12] = 3.853, *p* = 0.002, *t* test for independent samples; Fig. 6i, j), suggesting that TAT-inducible *BAF170cKO_Nestin-CreER* mice showed somewhat less proactive memory interference between the original and current platform position [53]. The reduced proactive memory interference of the inducible BAF170cKO during early stages of reversal learning is likely to be due to a weaker memory for the original platform position. Consequently, the inducible *BAF170cKO* mice showed inferior performance, as compared to the controls, on the late stages of reversal learning on days 5–8 (mean escape latency: T[12] = 2.054, *p* = 0.063, mean swimming distance: T[12] = 2.175, *p* = 0.050, *t* test for independent samples; Fig. 6k, l). No significant genotype differences were found for the reversal learning probe test (time spent in target quadrant: T[12] = 1.090, *p* = 0.297; number of crossings: T[12] = 0.574, *p* = 0.577, *t* test for independent samples; Fig. 6g, h).

In sum, these finding suggest that the cortex-specific knockout of *BAF170* during embryonic and later stages of hippocampal development (*BAF170cKO_hGFAP-Cre* mice) impairs the accuracy of place responding [51], possibly due to impaired stability of hippocampal place fields [52]. In contrast, complete ablation of *BAF170* at the age of ∼6 weeks (inducible *BAF170cKO-NESTIN-CreER* mice), leads to a moderate impairment in the hidden platform task, reduced proactive memory interference during early stages of reversal learning, and impaired spatial learning during the late stages of the reversal test.

## Discussion

Growing evidence indicates that epigenetic mechanisms control a variety of processes in the developing mammalian brain, such as fate-choice decisions of NSC/progenitor cells during development, as well as maintaining neurogenesis in adult brain niches, the anterior SVZ of the forebrain and SGZ of DG, throughout the life [4, 5]. Recent findings also suggest that epigenetic mechanisms of gene regulation (e.g., histone acetylation, DNA methylation) could be involved in establishment of long-term memory formation [54, 55]. The BAF chromatin remodeling complexes utilize the energy of ATP to disrupt the nucleosome DNA contacts, controlling the unpacking of the genomic DNA and its associated proteins, thereby regulating fundamental cellular processes. The combinatorial assembly of 14 BAF subunits of mammalian BAF complexes contributes to rather diverse functions under different contexts [56]. The different subunits interact with specific TFs and cofactors which is the basis for a great diversity of BAF target genes and chromatin remodeling activities at particular developmental stages and/or physiological conditions. Until recently, epigenetic mechanisms through chromatin remodeling have been studied mostly in the context of neuronal development in ES cells in vitro. Previously, we presented vivo data that during the early cortical neurogenesis (E10.5–E14.5), a Brm-based BAF complex timely represses the indirect mode of neurogenesis through a mechanism that involves an interaction between the BAF170 subunit and TF Pax6 [15].

Cognitive deficits may result from developmental abnormalities or could be also due to a role of chromatin remodeling during adult neurogenesis. Interestingly, even heterozygous mutations in different subunits of the BAF complex have been shown to cause similar intellectual impairments seen as in autism and schizophrenia [57]. The first evidence that BAF complexes could play a role in adult long-lasting forms of synaptic plasticity and memory was provided for BAF53b subunits whose deletion or overexpression caused severe long-term memory deficits [58].

In the present study, we generated hGFAP-Cre and TAM-induced Nestin-Cre-driven BAF170-deficient mice models to study the effect of late developmental or restricted postnatal elimination of BAF170 function in developing and postnatal hippocampus/SGZ of DG, respectively. This allowed us to disclose a role for BAF170 beyond neuronal development. The SGZ within the inner layer of DG of the adult brain constitutes a self-renewal neurogenic niche [3, 4, 43] for generation of postnatal NSCs. During normal development, age-related decline of neurogenesis includes loss of NSCs through a decrease in their proliferative capacity and differentiation, as well as *via* reduction in neuronal fate commitment [28, 43, 59, 60]. Characterization of both transgenic mice lines revealed that deletion of BAF170 in Hi-progenitors mostly during latest (E17.5 – E18.5) and postnatal neurogenesis *(BAF170cKO_hGFAP-Cre)* or the restricted deletion of BAF170 in adult NSC in SGL of DG (*BAF170cKO_Nestin-CreER)* leads to ectopic distribution and diminished proliferation of RGL cells within the DG blade, thus diminishing the pool of NSCs. In adult Hi, upon exiting their quiescent state, the NSCs undergo asymmetric divisions to produce dividing progeny that differentiate into neurons, while later converting into mature astrocytes [61, 62]. Thus, diminished production of new neurons in adult Hi may include depressed proliferative capacity of NSCs or reduced neuronal lineage commitment. Interestingly, in both *BAF170cKO-hGFAP-Cre* and *BAF170cKO-Nestin-CreER* young mutants (1 month old), the RGLs in DG appears to undergo a more intensive premature astrocytic differentiation, compared to controls, leading to almost complete depletion of NSC in adult DG (4 months old) in the mutant mice. The phenotype is consistent with studies showing that the amount of age-dependent disposable stem cells in the DG is partially dependent on efficient terminal differentiation of RGL cells to astrocytes [28, 43].

It is still unclear how the BAF complex controls astrocyte differentiation. During adult neurogenesis in the forebrain SVZ, a Brg1-based BAF complex and TF Pax6 control cell-fate decision (neuron/astroglia) [24]. Indeed, in conjunction with TF Pax6, the Brg1-based BAF complex regulates downstream effectors (*Pou3f4, Sox11, Nfib*) that normaly drive neurogenesis over gliogenesis [24]. Here, we demonstrated that Brg1-based BAF complex is also active in the adult SGZ of DG, and its BAF170 subunit is expressed in the astrocyte cell lineages (in GFAP+/Sox2+, GFAP+/S100β+, and GFAP+/Nestin− cells). In the postnatal Hi, TF Pax6 is expressed in GFAP+ radial glial progenitors extending long processes throughout DG [63]. Diminished Pax6 dosage as seen in heterozygous *Small eye* rats (*rSey+/−)* causes defect in maintenance of the RGL early progenitors in SGZ and a shift to the fate of late progenitors that show abnormal morphology (with almost missing long processes), and ectopic location in DGs [63]. Moreover, NSCs from *rSey/+* adult brains generate astrocytes in an excess [64]. The similarity of the progenitor phenotype in BAF170 (this study) and Pax6 loss-of-function strongly suggest that upon ablation of BAF170, a defect in the cooperation between BAF170 subunit of the remodeling complex and TF Pax6 in SGZ could account for enhanced astrogenesis. In support of such a scenario, at least 3 Pax6 direct target genes are involved in neuronal vs. glial fate acquisition, *Ngn2, Hmga2*, and *Pten* [65–67]. In a further support, Pten deletion in adult hippocampal SGZ results in a similar accelerated differentiation toward the astrocytic lineage and depletion of the NSC pool [68] as reported here for *BAF170LOF*. Nevertheless, further experimentations are needed to directly support such a scenario.

The Hi plays a crucial role for spatial learning and memory [69, 70]. Upon overexpression of TLX (an orphan nuclear receptor) in the SGL of DG, the mice exhibit increased memory acquisition and retention [71], whereas *TLXKO* mice exhibited learning impairment and further behavioral phenotypes [6, 72]. In the present study, we showed that conditional knockout of *BAF170* via hGFAPCre-driven recombination during latest stages of Hi development and postnatal period induces an impairment in the accuracy of place responding [51]. Since poor spatial navigation in rodents is associated with an impaired synaptic plasticity of hippocampal place cells and an impaired stability of their place fields [73–75], this behavioral phenotype could be a consequence of both, a mild defect of neurogenesis in Hi-proper as a potential source of adult place cell supply [76–78], together with deficiency of adult neurogenesis and cell differentiation in SGZ of DG. Remarkably however, upon restricted elimination of BAF170 expression in the SGL of adult *BAF170cKO_NESTIN-CreER* mice (4 month of age), we found a moderate spatial learning impairment during the in hidden platform task and a more severe spatial impairment after being challenged with a second spatial problem during the reversal learning test. This impairment in spatial learning is assumed to be indeed related to impaired adult Hi-neurogenesis [79–81].

In conclusion, our behavioral results suggest that BAF170-deficiency is not only important for the accurate development of the neurobiological substrates that underlie learning and memory performance (possibly accounting for the place responding deficits observed in the *BAF170cKO_hGFAP-Cre* mice), but also seems to be involved in the process of learning and memory formation in the adult animal with normally developed brain structures (as evidenced by impairments in reversal learning in the inducible *BAF170cKO-NESTIN-CreER* mice). These findings suggest that BAF complexes play important roles for normal brain development, and are also relevant for learning and memory formation *per se*. In this line of evidence, deficiency of neuron-specific subunit BAF53b from conception onwards or neuron-specific BAF53b mutation causes object memory deficits concomitant with impaired hippocampus-dependent long term potentiation in mice [58]. However, it should be noticed that the spatial learning deficit during the reversal learning test could be also due to impaired cognitive flexibility and adaptive behavior that is known to be associated with an impairment of the functioning of the prefrontal cortex [82, 83]. Further experimentations are necessary to determine whether the inducible *BAF170cKO-Nestin_Cre-ER* mice show impaired synaptic plasticity in the prefrontal cortex concomitant with impairments in reversal learning [84–86].

Interestingly, *BAF170cKO* mutant mice as shown in this work and *TlxKO*mutants described earlier [6] exhibit similar deficits in Hi-dependent spatial learning and memory. In both cases mild differences were observed regarding the learning curve (escape distance) and the number of visits to the virtual platform in the probe trial. Moreover, performance in contextual fear conditioning—another Hi-dependent learning and memory paradigm—was not impaired in both *BAF170cKO* and *TlxKO* mutant mice. These findings do not contradict our results of the Morris water maze task, since this test is much more complex and requires spatial navigation, in contrast to contextual fear conditioning, which is based on simple recognition of the training context. In addition, both BAF170 and TLX appear to be essential for the maintenance and positioning of RGL progenitors in the DG, and correct astrogliogenesis[6, 7, 20, 48]. In future studies, it would be interesting to investigate whether BAF170 acts as an upstream regulator of TLX or a factor that interacts with TLX in adult neurogenesis and learning/memory function.

## Conclusions

During the development of the CNS, different arrangement of the BAF subunits of the BAF complex controls neural stem/progenitor proliferation and initiation of cell differentiation [13, 56, 87]. Here, we have shown that elimination of BAF170 subunit function during latest stage of corticogenesis and in the postnatal brain produced decreased proliferation and mislocation of RGL cells in DG, as well as a premature astrocytic differentiation of NSCs. Moreover, our findings highlight a novel function of the BAF-chromatin remodeling in regulation of adult brain neurogenesis, involved in spatial learning and memory.

## Electronic Supplementary Material

Below is the link to the electronic supplementary material.

Table S1 Statistical analyses (XLSX 67 kb)

ESM 1 (PDF 1.48 mb)

## Abbreviations

Hi: Hippocampus
DG: Dentate gyrus
RGL: Radial glial-like cell
CNS: Central nervous system
SGZ: Subgranular zone
GL: Granular layer
NSC: Neural stem cell
TFs: Transcription factors
SWI/SNF: SWItch/Sucrose NonFermentable
BAF: Brg1/Brm-associated factor
ESCs: Embryonic stem cells
cKO: Conditional knockout
RGPs: Radial glial progenitors
IP: Intermediate progenitor
TAM: Tamoxifen
IHC: Immunohistochemistry
