# Supplementary material for: Ablation of BAF170 in Developing and Postnatal Dentate Gyrus Affects Neural Stem Cell Proliferation, Differentiation, and Learning
Source: Mol Neurobiol. 2016 Jul 8;54(6):4618–35. doi: 10.1007/s12035-016-9948-5 (PMC5509785; doi:10.1007/s12035-016-9948-5)
Supplement: Supplementary file 2 — (PDF 1.48 mb) [file 12035_2016_9948_MOESM2_ESM.pdf]

‘

Supplementary Material

Supplementary Experimental Procedures

Behavioral testing

Rotarod: This test examines motor coordination and balancing performance. It consisted of a horizontal drum (Ugo Basile, Comerio, Varese, Italy), that was accelerated from 4 to 40rpm in the course of 5min. Mice were placed individually on the drum and the latency of falling off the drum was recorded. To assess motor learning, the rotarod test was repeated 24h later.

Open field: Exploratory activity in a novel environment was tested in a circular grey Perspex arena (diameter: 120cm, height: 25cm). Mice were placed in the center and allowed to explore the open field for 7min. The behavior was recorded by a PC-linked overhead video camera. Viewer2 software (Biobserve GmbH, Bonn, Germany) was used to calculate running velocity, distance traveled, and time spent in the central, intermediate or peripheral zones of the open field.

Morris water maze: Spatial learning and memory was assessed in a mouse version of the water maze [1]. A large circular tank (diameter 1.2m, depth 0.4m) was filled with opaque water (25±1˚C, depth 0.3m) and the escape platform (10x10cm) was submerged 1cm below the surface. The swim patterns were monitored by a computer and the video-tracking system Viewer2 (Biobserve GmbH, Bonn, Germany). The escape latency, swim speed, path length, and trajectory of swimming were recorded for each mouse. During the first 2 days, mice were trained to swim to a visible platform (visible platform task) that was marked with a 15cm high black flag and placed pseudo-randomly in different locations across trials (non-spatial training). The extra-maze cues were hidden during these trials. After 2 days of visible platform training, hidden platform training (spatial training) was performed. For 8 days, mice were trained to find a hidden platform (i.e. the flag was removed) that was located at the center in1 of the 4 quadrants of the pool. The location of the platform was fixed throughout testing. Mice had to navigate to the platform using extra-maze cues that were placed on the walls of the testing room. Every day, mice received4 trials with an inter-trial interval of 5min. The

mice were placed into the pool facing the side wall randomly at 1 of 4 start locations and allowed to swim until they find the platform, or for a maximum of 90s. A mouse that failed to find the platform within 90s was guided to the platform. The mouse then remained on the platform for 20s before being removed from the pool. The day after completion of the hidden platform training, a probe trial was conducted in order to determine whether the mice had developed a spatial bias for the former platform quadrant. The platform was removed from the pool and the mice were allowed to swim freely for 90s. The percentage of time spent in each quadrant of the pool as well as the number of times the mice crossed the former position of the hidden platform were recorded. A reversal learning test that is thought to measure adaptive behavior and cognitive flexibility was initiated one day after the hidden platform probe trial. The experimental procedure was identical to the one used for the hidden platform training with the exception that the escape platform was moved from the original position to a neighboring quadrant.

References

1. Morris, R., *Developments of a water-maze procedure for studying spatial learning in the rat.* J Neurosci Methods, 1984. **11**(1): p. 47-60.
2. Tuoc, T.C., et al., *Chromatin regulation by BAF170 controls cerebral cortical size and thickness.* Developmental Cell, 2013. **25**(3): p. 256-69.

Supplementary Figures

Figure S1. The integration of BAF170 into Brg1-dependent BAF complex and expression of BAF170 in IP progenitors and mature neurons in adult hippocampus.

(A) Double-label IHC using anti-Brm (red) and anti-pHH3 (green) antibodies at E10.5, E12.5 and E15.5. Similar to BAF170 [2], mitotically active cortical progenitors express Brm at the apical surface of the VZ beginning at about

‘

E12.5, but lose expression around E15.5. **(B)** Immunoprecipitation assays of hippocampal tissue from two-months old mice indicate that BAF170 interacts with Brg1. Western blot analysis of lysate from Immunoprecipitation with BAF170 antibody or IgG as negative control. Results of Western blot analyses using an anti-Brg1antibodyshowthatBAF170 antibody, but not IgG (negative control), co-purifies Brg1. Input is 5% of the amount used in immunoprecipitation assays. **(C)** Double IHC analyses with BAF170 antibody (in red) and antibodies specifically labeling neuronal progenitors (Mash1, green), or mature neurons (NeuN, green) indicate that in DG, BAF170 is expressed in mature neurons, but is absent in the neuronal progenitors (Mash1+, empty arrows and MCM2+, Tbr2+ empty arrows in Fig. 1F/G). Note that in DG most BAF170+cells are immune reactive with NeuN antibody (filled arrow), but some of BAF170+cells are negative with NeuN in SGZ (empty arrows). Scale bars = 20μm.

Figure S2. Characterization of conditional deletion of BAF170 in vivo through Cre recombination via hGFAP-Cre line.

(A) Images show a double IHC analysis of cortical cross sections at E14.5 with antibodies for Cre recombinase (green) and BAF170 (red) in control and *BAF170cKO_hGFAP-Cre* brains. Although that at this stage the hGFAP-Cre line drives a high Cre-recombinases activity in cortical VZ, the expression of BAF170 is largely preserved in *BAF170cKO*comparedtocontrolmice.

(B, C) Images from IHC analysis with BAF170 antibody (green) on coronal sections from rostral cortex at 18.5 (B) and hippocampus at P1 (C) show a loss of BAF170 expression in *BAF170cKO_hGFAP-Cre*mutantsat P1 (C), but not at E18.5 (C).

‘

(D)IHC analysis with an anti-BAF170 antibody on coronal sections from 1.5-months old wild type and transgenic brains show a complete loss of BAF170 expression in *BAF170cKO_hGFAP-Cre* mice in both Hi and DG (D). DAPI staining (in blue) reveals a diminished size of Hi proper and DG upon deletion of BAF170.

(E) IHC with pyramidal neuronal marker Ctip2 (green) reveals a lower density of Ctip2+ neurons in CA1, CA2 fields and DG in *BAF170cKO_hGFAP-Cre* as compared to the control mice.

(F) IHC analyses of *BAF170cKO_hGFAP-Cre* cortices at E18.5 and hippocampus at 1.5 -months old with an anti-Casp-3 antibody indicates that loss of *BAF170* function does not increase cell death.

Scale bars = 250μm.

Figure S3. BAF170 is involved in maintenance of RGL cells and astrogenesis in the postnatal DG.

The images show triple IHC analysis of the expression of Nestin (red), GFAP (green) and BLBP (magenta) on coronal sections of Hi from 1.5-month old control and transgenic mice, as indicated. The middle panels shows images at a higher magnification***.*** Note that compared to control, DGs in the mutant is more abundantly populated with GFAP+/BLBP+ cells with astrocytic morphology, expressing Nestin only at a low level or showing virtual absence of Nestin immunostaining (empty arrows). In contrast, in the control brain, the extended radial fibers from the RGL cells are immunoreactive with Nestin, GFAP and BLBP antibody.

Figure S4. Expression of postnatal/adult NSC TLX in DG of 2.5-months old *BAF170cKO* mice.

(**A**) Images showed TLX immunostaining in theof 2.5-months old*BAF170cKO* and control mice.

‘

(B) Quantification comparing the number of TLX+ cells in the DG of*BAF170cKO* and control mice.

Values are presented as mean ± STD (n=12).

Figure S5. Expression of MCM2 (neuronal progenitor marker) and GS (astrocyte marker) in DG of young and adult *BAF170cKO* mice.

(A, B) Images show IHC analyses of DG tissues of young (1 month old) and old (4 months old) mice with antibodies specifically labeling proliferating progenitors (Mcm2) and astrocytes (GS).

(C, D) Diagrams show statistic alanalysis data comparing the number of Mcm2+ proliferating progenitors (in the selected areas in A), GS+ astrocytes (in the selected areas in B) at indicated ages.

Values are presented as mean SEM (n=12). Scale bars= 20μm.

Figure S6. BAF170 deficiency has no detrimental effect on motor coordination and exploratory activity.

Conventional and TAM-inducible *BAF170cKO_Nestin-CreER*mice showed no changes in the rotarod test for motor coordination and balancing function **(A, D).** Similarly, exploratory activity in the open field test was normal in both conventional **(B, C)** and TAM-inducible *BAF170cKO_Nestin-CreER* mice (**E, F**). Data is presented as mean ± SEM. Numbers given in the bars refer to corresponding sample sizes.

Table S1: statistical analyses

‘

Figure S1

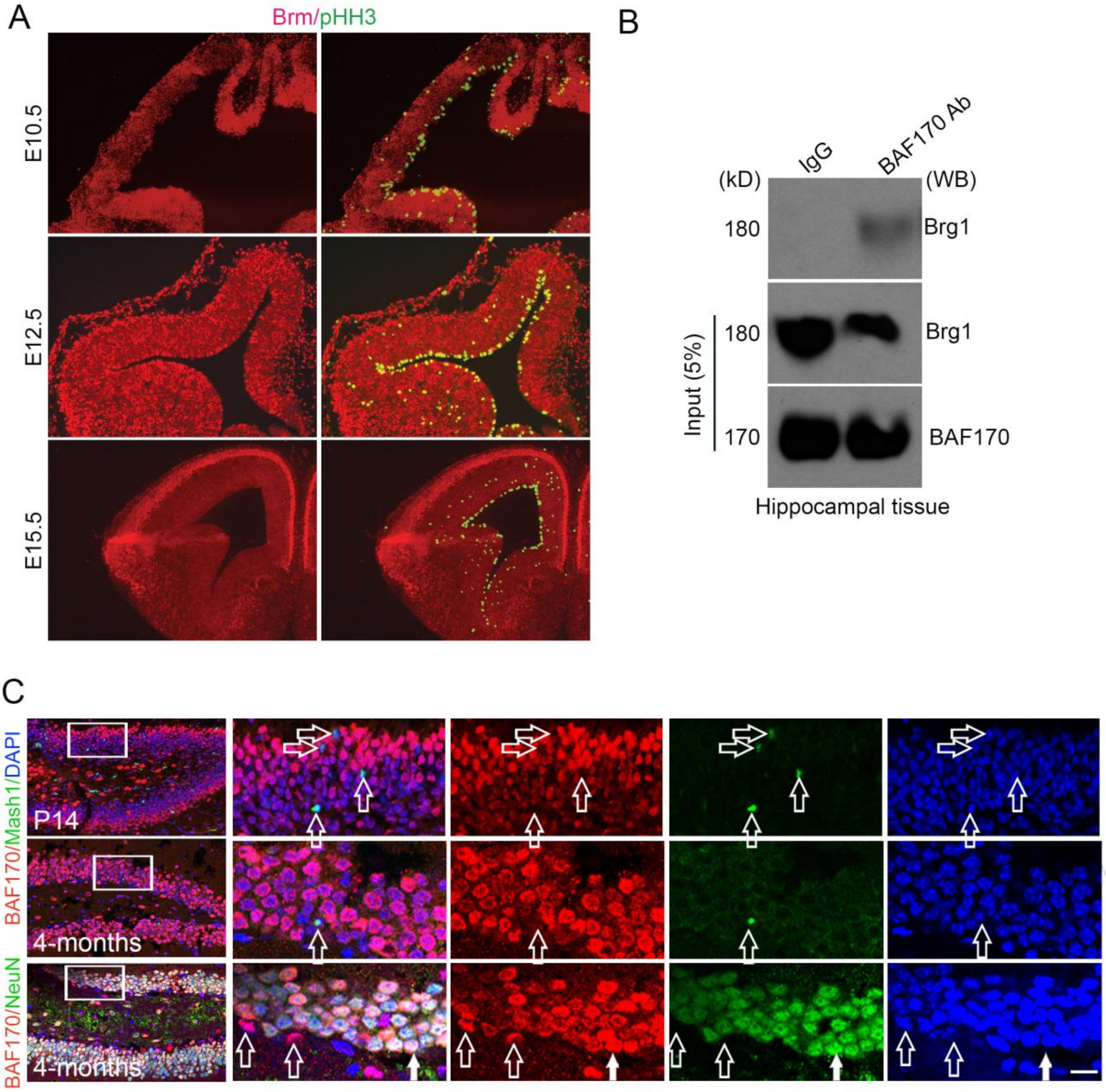
A
Brm/pHH3
E10.5
E12.5
E15.5
B
IgG
BAF170 Ab
(kD)
(WB)
180
Brg1
Input (5%)
180
Brg1
170
BAF170
Hippocampal tissue
C
BAF170/Mash1/DAPI
P14
4-months
BAF170/NeuN
4-months

‘

Figure S2

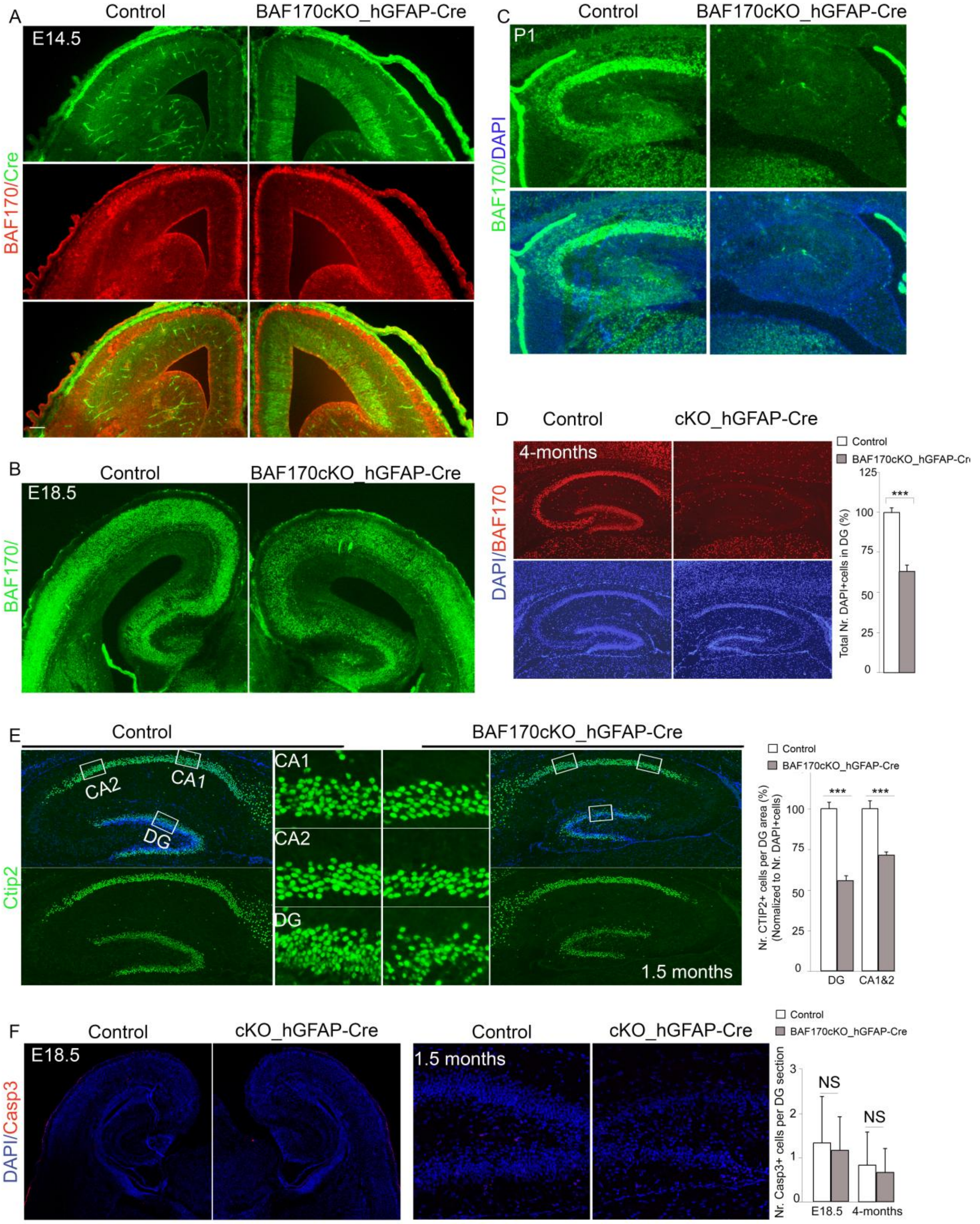

A
Control
BAF170cKO_hGFAP-Cre
E14.5
BAF170/Cre
B
Control
BAF170cKO_hGFAP-Cre
E18.5
BAF170/
C
Control
BAF170cKO_hGFAP-Cre
P1
BAF170/DAPI
D
Control
cKO_hGFAP-Cre
4-months
DAPI/BAF170
Control
BAF170cKO_hGFAP-Cre
Total Nr. DAPI+cells in DG (%)
***
E
Control
BAF170cKO_hGFAP-Cre
CA2
CA1
DG
Ctip2
1.5 months
Control
BAF170cKO_hGFAP-Cre
Nr. CTIP2+ cells per DG area (%)
(Nomalized to Nr. DAPI+cells)
DG
CA1&2
F
Control
cKO_hGFAP-Cre
E18.5
DAPI/Casp3
Control
cKO_hGFAP-Cre
1.5 months
Control
BAF170cKO_hGFAP-Cre
Nr. Casp3+ cells per DG section
NS
NS
E18.5
4-months

‘

Figure S3

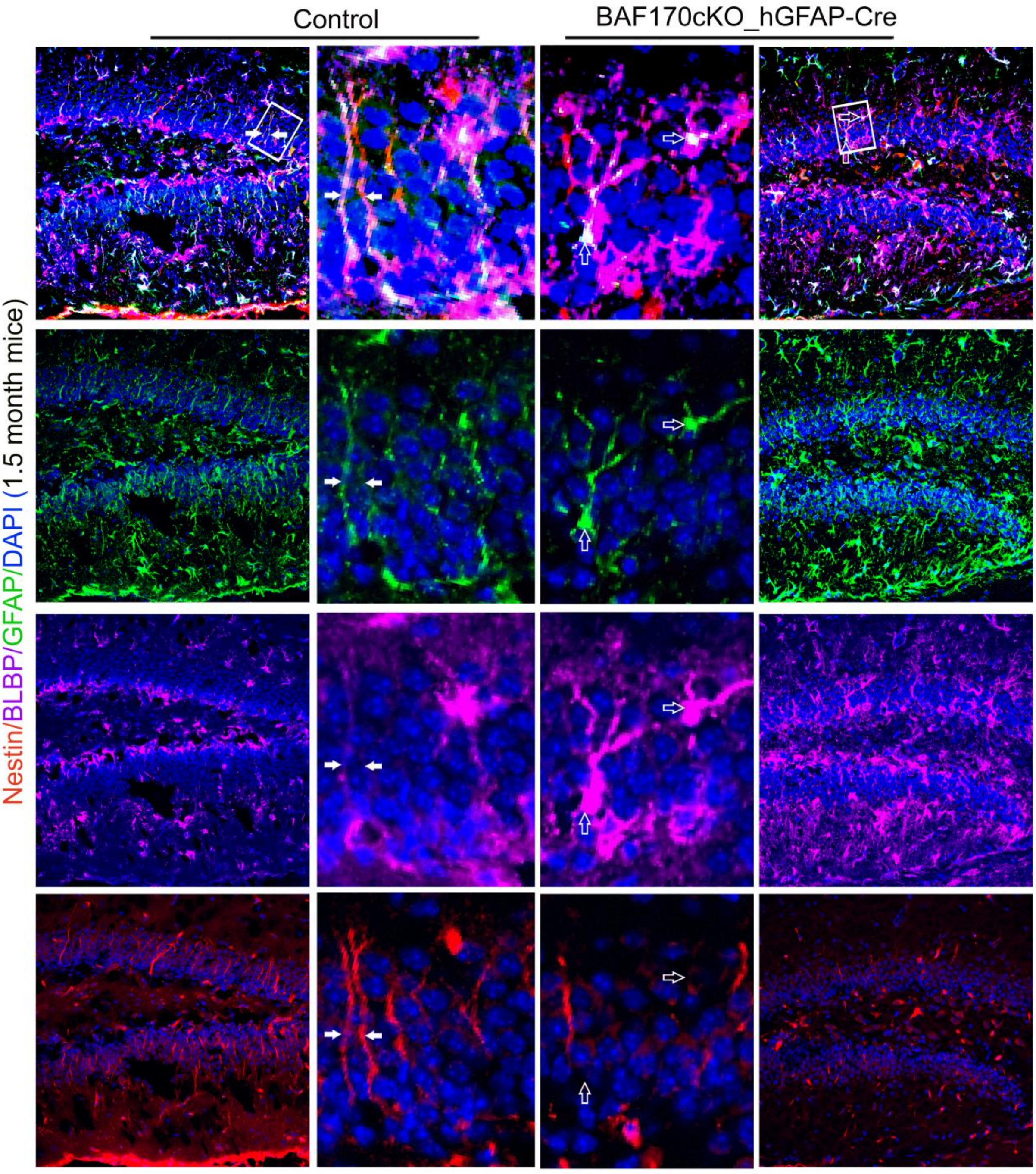

Control
BAF170cKO_hGFAP-Cre
Nestin/BLBP/GFAP/DAPI (1.5 month mice)

‘

Figure S4

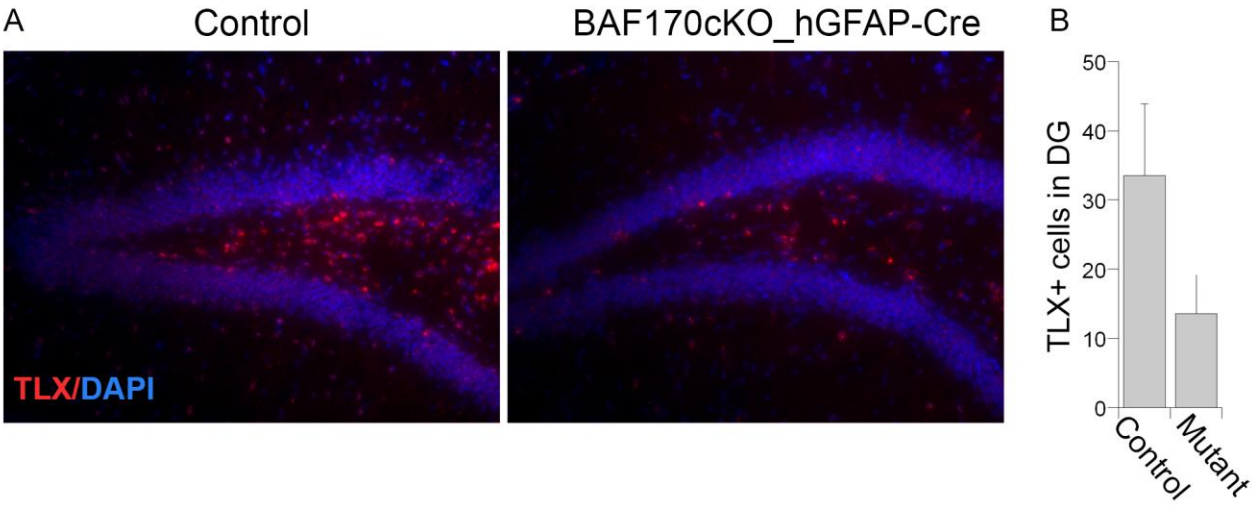
A
Control
BAF170cKO_hGFAP-Cre
TLX/DAPI
B
TLX+ cells in DG
50
40
30
20
10
0
Control
Mutant

‘

Figure S5

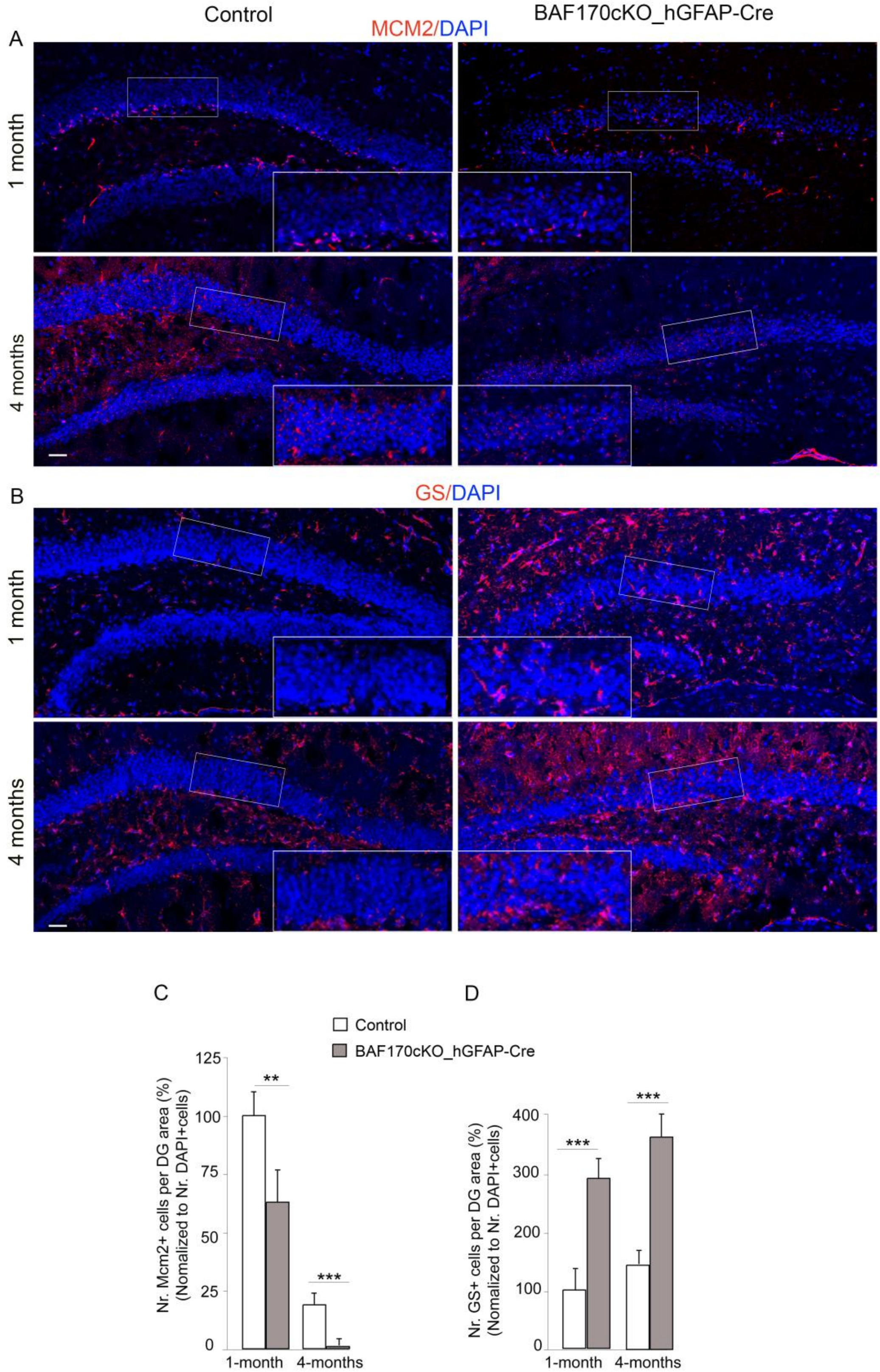
Control
MCM2/DAPI
BAF170cKO_hGFAP-Cre
A
1 month
4 months
B
GS/DAPI
1 month
4 months
C
D
Control
BAF170cKO_hGFAP-Cre
Nr. Mcm2+ cells per DG area (%)
(Nomalized to Nr. DAPI+cells)
125
100
75
50
25
0
**
***
1-month
4-months
Nr. GS+ cells per DG area (%)
(Nomalized to Nr. DAPI+cells)
400
300
200
100
0
***
***
1-month
4-months

‘

Figure S6

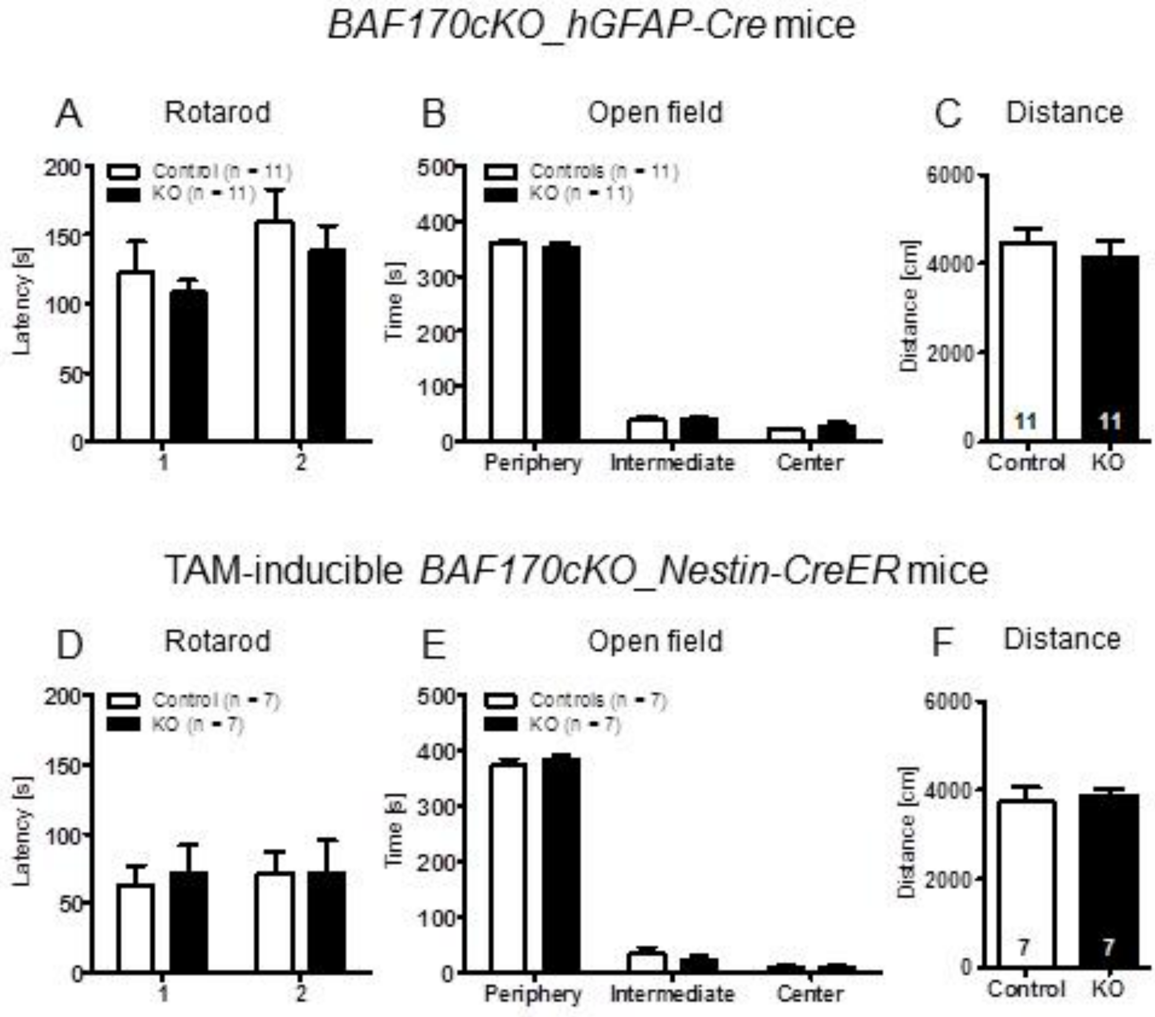
BAF170cKO_hGFAP-Cre mice
A Rotarod
Control (n = 11)
KO (n = 11)
Latency [s]
B Open field
Controls (n = 11)
KO (n = 11)
Time [s]
Periphery
Intermediate
Center
C Distance
Distance [cm]
11
11
Control
KO
TAM-inducible BAF170cKO_Nestin-CreER mice
D Rotarod
Control (n = 7)
KO (n = 7)
Latency [s]
E Open field
Controls (n = 7)
KO (n = 7)
Time [s]
Periphery
Intermediate
Center
F Distance
Distance [cm]
7
7
Control
KO
